# ITCH nuclear translocation and H1.2 polyubiquitination negatively regulate the DNA damage response

**DOI:** 10.1093/nar/gky1199

**Published:** 2018-12-04

**Authors:** Lufen Chang, Lei Shen, Hu Zhou, Jing Gao, Hangyi Pan, Li Zheng, Brian Armstrong, Yang Peng, Guang Peng, Binhua P Zhou, Steven T Rosen, Binghui Shen

**Affiliations:** 1 Department of Cancer Genetics and Epigenetics, Beckman Research Institute, City of Hope, Duarte, CA 91010, USA; 2 Department of Analytical Chemistry, Shanghai Institute of Material Medical Science, Chinese Academy of Sciences, Shanghai, China; 3 Department of Developmental and Stem Cell Biology, Beckman Research Institute, City of Hope, Duarte, CA 91010, USA; 4 Department of Clinical Cancer Prevention, The University of Texas MD Anderson Cancer Center, Houston, TX 77030, USA; 5 Department of Molecular and Cellular Biochemistry, Markey Cancer Center, University of Kentucky, College of Medicine, Lexington, KY 40506, USA; 6 Department of Hematology and Hematopoietic Cell Transplantation, Beckman Research Institute, City of Hope, Duarte, CA 91010, USA

## Abstract

The downregulation of the DNA damage response (DDR) enables aggressive tumors to achieve uncontrolled proliferation against replication stress, but the mechanisms underlying this process in tumors are relatively complex. Here, we demonstrate a mechanism through which a distinct E3 ubiquitin ligase, ITCH, modulates DDR machinery in triple-negative breast cancer (TNBC). We found that expression of a nuclear form of ITCH was significantly increased in human TNBC cell lines and tumor specimens. Phosphorylation of ITCH at Ser257 by AKT led to the nuclear localization of ITCH and ubiquitination of H1.2. The ITCH-mediated polyubiquitination of H1.2 suppressed RNF8/RNF168-dependent formation of 53BP1 foci, which plays important roles in DDR. Consistent with these findings, impaired ITCH nuclear translocation and H1.2 polyubiquitination sensitized cells to replication stress and limited cell growth and migration. AKT activation of ITCH-H1.2 axis may confer TNBC cells with a DDR repression to counteract the replication stress and increase cancer cell survivorship and growth potential.

## INTRODUCTION

Breast cancer (BC) is the most frequently diagnosed type of cancer in women worldwide (1). Approximately 30% of women initially diagnosed with early-stage disease will ultimately develop metastatic lesions, and nearly half of all BC patients develop distant metastatic disease after chemotherapeutic and/or hormonal agent treatment (2). Unfortunately, current clinical strategies fail to adequately treat metastatic disease, and the mechanisms underlying BC metastases remain poorly understood.

Patients with basal-like triple-negative BC (TNBC), the most aggressive BC subtype (1), have high rates of recurrence and distant metastases, which exhibit high levels of DNA replication stress (3). DNA replication stress and DNA damage induce the formation of aberrant DNA structures that trigger the DNA damage response (DDR) signaling pathway (4,5). DDR typically leads either to DNA repair, or in the case of irreparable damage, to apoptosis or senescence (6,7). When oncogenes induce persistent DNA replication stress, high mutation rates, and severe genomic instability; tumor cells may downregulate or acquire faulty DDR mechanisms through genetic and epigenetic alterations that support continued survival despite of potential genomic damage (6,7). Thus, the dysregulation of genes that encoding DDR machinery and genes involved in DNA repair have been associated with tumor development, progression, metastasis, malignancy grade, and patient prognosis and survival across many cancers (4,5,8,9). Therefore, interventions to restore DDR signaling to promote tumor cell death could potentially serve as efficacious cancer therapies.

In response to DNA damage, such as double strand breaks (DSBs), histone H2AX is phosphorylated (to γH2AX) by PI3K-like kinases (PIKKs), which initiates the recruitment of many DDR factors, such as MDC1, which activate cell cycle checkpoints and DDR and can serve as scaffold proteins for the recruitment other downstream DDR factors (2,3,6). The ubiquitin (Ub)-dependent DNA damage signaling cascade is an important regulatory mechanism of the DDR (10). Polyubiquitinated histone H1 was recently shown to serve as an important signaling intermediate for the DSB repair process that depends on the E3 Ub ligases RNF8 and RNF168 (11,12). Whether the activity of polyubiquitinated histone H1 and RNF8/RNF168-dependent DDR events are negatively regulated in aggressive tumors, however, has not yet been explored.

ITCH is a member of the E6-AP carboxyl terminus (HECT) subfamily of E3 Ub ligases (10). ITCH ubiquitination (Ubn) controls distinct physiological processes in normal cells, including DDR, T-cell differentiation, the immune response, and cell death (13,14). ITCH gene copy numbers are amplified in anaplastic thyroid carcinoma (15) and in several other human malignancies, including BC, according to the Oncomine database. In the current study, we provide the first evidence that ITCH can function as an epigenetic regulator of the DDR that is overexpressed in BC cell lines and tumors. We define a mechanism through which poly-Ubn of H1.2 by nuclear AKT-activated ITCH suppresses cellular DDR signaling to counteract replication stress in TNBC cells. The PI3K/AKT pathway is a major pathway that leads to tumor proliferation in BC (16). Aberrant activation of this pathway, which occurs due to loss of the lipid phosphatase PTEN or activating mutations in the PIK3CA gene, was identified in a large series of TNBC patient samples (17). AKT activation of ITCH may confer TNBC cells with a DDR repression mechanism to counteract the replication stress constitutively induced by PI3K/AKT signaling, thus increasing cancer cell survivorship and growth potential. Tumor invasion and metastasis are direct causes of cancer mortality and represent the central clinical challenge of solid tumor oncology. Mapping the signaling cascades essential to the metastatic program, such as the PI3K/AKT/ITCH/H1.2 pathway, will enable the development of more efficient treatment options.

## MATERIALS AND METHODS

### Human clinical samples

Tissue microarrays (TMAs) of 282 invasive BC cases with clinical data, including ER/PR/HER2 status, grades, and stages, were collected from resected breast tumors of patients with informed consent and institutional IRB approval from the Markey Cancer Center Biospecimen Tissue and Procurement Shared Resource Facility (P30CA177558) at the University of Kentucky, Lexington. TMAs containing 100 cases of BC with normal tissue control specimens (BR1002a) and 50 cases of invasive ductal carcinoma and matched metastatic invasive ductal carcinoma of lymph nodes from breast (BR1005) were purchased from US Biomax, Inc.

### Cell culture

HEK293T cells were maintained in DMEM containing 10% fetal bovine serum with antibiotic/antimycotic solution (Invitrogen). BC cell lines were cultured according to the manufacturer's protocol (ATCC). To establish stable knockdown of ITCH, stable clones of MDA-MB-231 cells transfected with ITCH shRNA were selected with puromycin (300 ng/ml) for 4 weeks. MDA-MB-231 clones with stable expression of GFP-tagged WT ITCH, S257A ITCH, WT-H1.2 or K46R-H1.2 were selected with G418 (3 mg/ml). To establish luciferase-transfected MDA-MB-231 stable cells, cells were transduced with 2 × 10^7^ lentiviral particles expressing firefly luciferase (Amsbio). Blasticidine (10 mg/ml) was added to select stably transduced cells. Maximum luciferase activity was detected using a microplate spectrofluorometer (Molecular Devices).

### Immunohistochemistry (IHC)

IHC was performed on tissue microarray (TMA) sections using an anti-ITCH primary antibody (BD Biosciences, 1:100, 2 h, 37°C) in the Pathology Core Laboratory at the Beckman Research Institute of City of Hope. Staining was visualized with 3,3′-diaminobenzidine (DAB) and hematoxylin counter staining. Digital images of IHC-stained TMA slides were obtained at 40× magnification (0.0625 μm^2^ per raw image pixel) using a Hamamatsu Nanozoomer 2.0-HT whole slide scanner and images were retrieved using NDP.view2 (Hamamatsu Photonics). ITCH expression in each entire TMA spot was quantified by a visual grading system based on the extent of staining. All scoring was performed by a single investigator blinded to the findings regarding other pathological stains and patient outcome. Only immunoreactivity in the nucleus was evaluated. Immunostaining for nuclear ITCH in each entire TMA spot was graded as follows: negative, no staining of cells; low, staining <5% of cells; medium, staining >5% and <50% of cells; high, staining >50% of cells.

### Immunoblotting (IB)

Cells were lysed in ice-cold buffer [150 mM NaCl, 10 mM Tris–Cl pH 7.4, 5 mM EDTA, 1% Triton X-100] supplemented with protease inhibitors (Sigma-Aldrich). The protein concentration of each sample was quantified using the Bio-Rad protein assay reagent (Cat#: 5000006, Bio-Rad). For detection of histone and histone modify cations, total histones were isolated using the Histone Extraction Kit (Cat#: ab113476, Abcam). Equal amounts of whole cell extracts or total histones were resolved by 4–15% or 15% SDS-PAGE and immunoblotted with specified antibodies. To detect the loading control including actin, GAPDH or histone H3 the blot was stripped and reprobed with an indicated antibody.

### Immunofluorescence (IF) staining, microscopy and foci quantification

Cells were grown on cover-slips (Fisherbrand, Cat #12-545-80) in 6- or 24-well dishes, fixed for 10 min with 4% paraformaldehyde, permeabilized for 10 min with PBS containing 0.3% Triton X-100 (PBS-T), and blocked for 1 h in 5% bovine serum in PBS. Primary antibodies were diluted in blocking buffer and incubated with fixed cells overnight at 4°C. Cells were washed and incubated with Alexa Fluor 488-, 555-, or 685-conjugated goat anti-mouse or -rabbit IgG (H+L) antibodies (Molecular Probes). Images were acquired using an Axio Observer Inverted Microscope System (Zeiss) or a laser scanning confocal microscope (Zeiss LSM 880 with Airyscan) in the Light Microscopy Digital Imaging Core at the Beckman Research Institute of City of Hope. For focus quantification experiments, nuclei were identified using the blue (4′,6-diamidino-2-phenylindole, DAPI) channels and foci were imaged using the green (Alexa Fluor 488) or red (Alexa Fluor 555) channels. Focus counting analysis was performed manually (by eye). To reduce potential bias of the manual counting analysis, focus quantification was validated by automatic counting using Image Pro image analysis software (Media Cybernetics Inc., USA). Acquired objects were classified as foci when their diameter was between 0.25 and 1.2 μm. Foci bigger than 1.2 μm were classified as clusters. Further cluster analysis was performed by determining the cluster size and the estimated number of foci contained within the area. For each sample, 30 to 100 cells were analyzed and different parameters, such as the number of selected cells, average foci number/cell, mean intensity, and number of foci-positive cells were calculated. Cells with ≥ 5 foci were counted as γH2AX focus-positive cells, and the percentage of γH2AX focus-positive cells in the range of 30–100 DAPI-stained cells was calculated. Data are presented for three independent experiments.

### Immunoprecipitation (IP)

Cells were lysed in buffer [50 mM Tris pH 7.5, 150 mM NaCl, 1% Nonidet P-40, 1 mM EDTA, and protease inhibitors]. Total cell lysates (1 ml) were incubated with 1 μg antibodies overnight at 4°C, followed by 2 h incubation with 50 μl protein G-conjugated beads (Thermo Fisher Scientific). Beads were washed with lysis buffer and the IP protein complexes were analyzed by IB.

### ITCH associated proteins analyzed by mass spectrometry (MS)

MDA-MB-231 cells were lysed in buffer [20 mM Tris–HCl, pH 7.3, 300 mM KCl, 0.2 mM EDTA, 0.5% Triton, 10% glycerol, 1 mM phenylmethylsulfonyl fluoride, 10 mM glycerophosphate, and 0.1 mM Na_3_VO_4_, and protease inhibitors]. Total protein (50 mg) was IP with anti-ITCH (1:1000; BD Biosciences) or unspecific mouse monoclonal IgG antibodies (I-1000; GeneTex Inc.) and 50 μl protein G-conjugated beads (Thermo Fisher), as above. Washed beads were separated by SDS-PAGE. Following Coomassie Blue Staining, protein bands were excised and digested. After separation on a reversed phase LC column, eluted peptides were analyzed on a MALDI-QIT-TOF-based mass spectrometer with electrospray ionization (Micromass/Waters). The MS/MS data were processed using Masslynx software (Micromass), and the MASCOT (Matrix Science) search engine was used to search the NCBI non-redundant database. Protein identifications were based on a minimum random probability score of 25 and with a mass accuracy of 0.1 Da.

### Plasmids, mutagenesis, transfection, shRNA

Full-length human ITCH cDNA was amplified from human Hela cDNA and cloned into the pcDNA3.1 (Xpress-tag, Invitrogen) and pET-32a(+) (Novagen) vectors. Ala substitution at S257 of ITCH and Arg substitution at K46 of H1.2 was performed using QuikChange II Site-Directed Mutagenesis Kit (Agilent Technologies). WT ITCH, S257A ITCH, WT H1.2, and K46R H1.2 cDNAs were cloned into pEGFP-C1 (Clontech), a gift from Dr. Yanzhong Yang (City of Hope). ITCH deletion mutants were generated by PCR and subcloned into Xpress-tag pcDNA3.1. ITCH shRNA and scrambled control shRNA were purchased from MISSION shRNA at Sigma-Aldrich. HEK293T or MDA-MB-231 cells were transfected using PolyJet DNA transfection Reagent (SignaGen). DDK-tagged H1.2, H1.3 and H1.4 plasmids were purchased from OriGene Technologies, Inc. pRK5-HA-ubiquitin-K63, pRK5-HA-ubiquitin-K48, and pRK5-HA-ubiquitin-K29 were purchased from Addgene.

### Subcellular fractionation

HEK293T or breast cancer cells were washed with ice-cold PBS, harvested by scraping or trypsinization, and collected in a hypotonic lysis buffer [20 mM HEPES pH 7.0, 10 mM KCl, 2 mM MgCl_2_, 0.5% NP-40, 1 mM Na_3_VO_4_, 1 mM phenylmethylsulfonyl fluoride, and protease inhibitors] for 10 min on ice. Cells were homogenized by 20 strokes in a tight-fitting Dounce homogenizer (Kontes disposable pestle with microtubes, Fisher Scientific). After centrifugation at 1300 × *g*, 4°C, and 5 min to sediment nuclei, the supernatant was transferred to a new tube and centrifuged at 13 000 × *g* for 20 min. The resulting supernatant was collected as a cytoplasmic fraction and transferred to a prechilled tube. The nuclear pellet was washed three times with hypotonic lysis buffer, resuspended, and periodically vortexed in nuclear extraction buffer [20 mM HEPES pH 7.9, 400 mM NaCl, 1 mM EDTA pH 8.0, 1 mM EGTA pH 7.0 and protease inhibitors] on ice for 30 min. After centrifugation at 13 000 × *g* for 10 min at 4°C, the nuclear lysates were collected and transferred into prechilled tubes.

### *In vitro* kinase assays

For kinase assays, endogenous kinase was IP from cells using specific antibodies according to the published protocol (18). Protein substrates were added to protein G conjugated-beads in buffer [25 mM MOPS pH 7.2, 12.5 mM β-glycerol phosphate, 20 mM MgCl_2_, 12.5 mM MnCl_2_, 2 mM EDTA pH 8.0, 5 mM EGTA and 0.25 mM DTT], with the addition of 20 mM ATP for cold reactions, and 10 mM ATP mixed with 10 μCi of [γ-^32^P] ATP for radioactive reactions. The reaction mixture was incubated at 37°C for 30 min, stopped by the addition of SDS loading buffer, followed by SDS-PAGE. Phosphorylated proteins were detected using autoradiography and protein levels were visualized using Coomassie blue staining.

### Generation of phospho-S257 ITCH antibody

Two peptides were used to generate a phospho-S257 (P-S257) ITCH antibody: C-SRPPRP(pS)RPPPPT-amide (phosphopeptide) and C-SRPPRPSRPPPPT-amide (non-phosphopeptide). These sequences showed homology with no other proteins but ITCH. The P-S257-ITCH antibody was generated by Life Technologies.

### MS analysis of ITCH phosphorylation by AKT

After *in vitro* phosphorylation by AKT, Coomassie blue-stained ITCH recombinant protein was excised from the gel. Gel fragments containing ITCH protein were subjected to proteolysis with trypsin. The tryptic peptides were then analyzed by nanoflow liquid chromatography tandem MS (LC-MS/MS) on an Orbitrap Elite (Thermo Scientific) interfaced with an EasyLC nano LC system using a self-packed column (75 μm × 150 mm; 3 μm ReproSil-Pur C18 beads, 120 Å, Dr Maisch GmbH) at a flow rate of 300 nl/min. The mass spectrometer was operated in data-dependent mode with each full MS scan (m/z 350-1800) followed by MS/MS for the 15 most intense ions with the parameters: ≥+2 precursor ion charge, 1 Da precursor ion isolation window, and 35 normalized collision energy of CID. The precursor and fragment ions were analyzed at 60 000 and 15 000 resolutions, respectively, and searched with Maxquant (version 1.5.1.0) against ITCH protein sequence with Rattus as the species. Variable modifications allowed were oxidation of M and phosphorylation S, T or Y. Data were filtered at a 1% false discovery rate at protein, peptide and phosphosite for high peptide confidence. The phosphopeptide sequence and site localizations were confirmed by manual inspection of the corresponding MS/MS spectra.

### In vitro ubiquitination assays

In vitro ubiquitination assay was performed according to the published protocol (19). Purified WT ITCH E3 ligase and H1.2 substrates were incubated with E1, UbcH7 E2, purified recombinant Itch-delta C2, and ubiquitin in reaction buffer [25 mM Tris–HCl, 100 mM NaCl, 1 mM DTT, 2.5 mM ATP and 4 mM MgCl_2_]. After incubation for 90 min at 30°C, the reactions were terminated by boiling in SDS loading buffer and resolved by SDS-PAGE gel.

### In vivo ubiquitination assay

In vivo ubiquitination assay was performed according to the published protocol (20) with the following modifications: Briefly, 293T cells were transfected with his-ubiquitin and other indicated plasmids for 36 h and harvested. To detect HA-tagged proteins, cell pellets resuspended at 4°C in lysis buffer (50 mM Tris–HCl at pH 7.4, 150 mM NaCl, 1 mM EDTA, 1% Triton X-100) containing protease inhibitors and 5 mM NEM were disrupted by sonication (2 × 10 s, 15% output, Virsonic 475 sonicator) and left on ice for 30 min. Lysates were centrifuged at 10 000 × *g* for 5 min twice, and soluble protein was quantified by BCA assay (Pierce). For immunoprecipitation of ubiquitinated HA-H1.2, lysate protein (500 μg) was diluted to 0.5 ml in RIPA buffer containing protease inhibitors. Lysates were precleared with protein G-Sepharose (ThermoFisher) beads for 1 h, and precleared supernatants were precipitated with anti-HA (3F10, Sigma) or anti-DDK (Origene) antibodies. Immune complexes recovered with protein G-Sepharose were washed six times with RIPA buffer and boiled in 6× sample buffer for 5 min. Denatured immune complexes were analyzed by western blotting and detected with antibodies to His (Cell Signaling) for Ubn. For detection of His-tagged ubiquitinated H1.2 species, 293T cells after transfection with the indicated plasmids for 36 h were harvested by denaturing buffer A (6  M guanidine–HCl, 0.1 M Na_2_HPO_4_/NaH_2_PO_4_, 10 mM imidazole pH 8). Pre-washed Ni-NTA agarose beads (QIAGEN) were incubated with cell lysates for 3.5 h to pull down his-ubiquitin and his-ubiquitin-conjugated proteins. Beads were then washed by buffer A and buffer T1 (25 mM Tris–Cl, 20 mM Imidazole pH 6.8) and analyzed by western blotting.

### Comet assay

Single-cell gel electrophoretic comet assays were performed following the manufacturer's protocol (Enzo Life Sciences). Briefly, MDA-MB-231 were collected, rinsed twice with ice-cold PBS, combined with LM Agarose at 37°C at a ratio of 1:10 (v/v), and immediately pipetted onto slides. For cell lysis, the slides were immersed in pre-chilled Lysis Solution at 4°C, for 30–60 min, then immersed in freshly prepared Alkaline Solution, pH >13, for 20–60 min at room temperature, in the dark. The slides were subjected to electrophoresis at 15 V for 25 min (0.6 V/cm), and stained with CYGREEN^®^ Nucleic Acid Dye for 30 min. Images were taken with a fluorescence microscope. Generally, a total of 50 cells in triplicate were analyzed per group. Comet tail length was measured using an ocular micrometer and DNA damage was calculated as: Comet tail length (μM) = maximum total length - head diameter.

### Purification of recombinant proteins

Full-length ITCH protein containing the C2 domain cannot be expressed in bacteria. Therefore, the ITCH recombinant proteins used in all experiments were truncated ITCH proteins without the C2 domain. Truncated ITCH constructs were generated by PCR as follows: ITCH (ITCH protein with C2 domain deletion, aa 10–102), WW (four WW domains, aa 326–511), HECT (HECT domain, aa 569–903). ITCH deletion and S257A mutant plasmids were transformed into BL21 (DE3)-competent cells (New England Biolabs), and proteins were expressed with 0.3 mM isopropyl bD-thiogalactopyranoside induction at 18°C overnight and purified according to the manufacturer's protocol (Novagen). Briefly, the cell pellet was lysed in buffer [50 mM tTis–Cl pH 7.5, 200 mM NaCl, 1.0% NP-40, 0.1% Na-deoxycholate, and protease inhibitors] on ice for 30 min, and the supernatant was collected by centrifugation at 11 000 × *g* for 10 min at 4°C. His-tagged proteins were precipitated using Ni-nitrilotriacetic acid resins (Clontech) with rotation at 4°C for 1 h. The resins were washed three times with lysis buffer and eluted with lysis buffer containing 150 mM imidazole. WT and K46R H1.2 proteins were expressed as HA-tagged recombinant proteins in 293T cells and purified using affinity chromatography according to the manufacturer's protocol (Thermo Fisher Scientific). Protein expression was confirmed by Coomassie blue staining and IB using anti-HA antibodies (1:1000) against HA-tag. Recombinant GST-tagged H1.2 proteins were purchased from Novus Biologicals.

### Nucleosome pull-down assays

HA-tagged WT or K46R H1.2 recombinant proteins before and after *in vitro* Ubn by ITCH were incubated with nucleosomes containing purified and refolded histone octamers and biotinylated 601DNA (EpiCypher) in binding buffer (20 mM HEPES, pH 7.9; 150 mM NaCl; 0.2 mM EDTA, 10% glycerol; 0.1% NP40; 1 mM DTT and complete protease inhibitors (Roche)) in the presence of streptavidin agarose (Thermo Fisher Scientific) for 18 h at 40°C. After five washes in binding buffer, the beads were collected and bound proteins were eluted in sample buffer and subjected to immunoblot analysis.

### Ionizing radiation (IR)

MDA-MB-231 cells were exposed to IR with total doses of 1 or 2 Gy using a CS-137 irradiator in the Animal Core Facility at the Beckman Research Institute of City of Hope.

### Animal model of breast cancer

All animal experiments were approved by the Institutional Animal Care and Use Committee at City of Hope and performed in the Animal Core Facility at the Beckman Research Institute of City of Hope. Female NSG mice, purchased from the Jackson Laboratory were injected in the fourth mammary fat pad with MDA-MB-231 cells (5 × 10^6^ cells/0.1 ml in HBSS) transfected with a scrambled shRNA (shControl) or shRNA targeting ITCH (shITCH) at the age of 8–10 weeks. Tumor volumes were monitored and bioluminescent *in vivo* imaging was performed weekly starting one week after tumor engraftment. Tumor volume was calculated using the following formula: volume (mm^3^) = *a*^2^ × *b*/2, where *a* is the tumor width in mm and *b* is the tumor length in mm. Bioluminescent *in vivo* imaging was monitored using a highly sensitive CCD camera mounted in a light-tight specimen box (IVIS-200TM, Xenogen, Caliper Life Sciences,). Animals were given the substrate d-luciferin potassium salt by intraperitoneal injection of 150 mg/kg in Dulbecco's phosphate buffered saline and then were anesthetized with 1–3% isoflurane gas. Ten min after the d-luciferin injection, mice were placed onto the warmed stage inside the light-tight camera box with continuous exposure to 1–2% isoflurane gas. Imaging acquisition time was from 1 s to 1 min, depending on the bioluminescence signal. Imaging and quantification of signals were obtained using the acquisition and analysis software Living Image V. 2.50 (Xenogen Corp.). To measure the intensities of the emitted light, the regions of interest (ROIs) were drawn over the emitted region of target signal (total p/s/cm^2^/sr).

### Real-time quantitative PCR (qPCR)

Total RNA was extracted from cells using RNAeasy (QIAGEN). cDNA was generated using SuperScript II (Invitrogen). The relative levels of mRNAs were determined by qPCR using Cyclophilin mRNA for normalization. Real-time qPCR was carried out using CFX96 Touch™ Real-Time PCR Detection System (Bio-Rad). Each PCR was performed in triplicate with the following profile: the first cycle at 95°C for 5 min, then 40 cycles at 95°C for 15 s and finally 60°C for 1 min (i.e. two-step protocol). Primer sequences of the tested genes are available upon request.

### DNA fiber assay

DNA Fiber assays were performed according to the previously published protocols (21) with the following modifications using the manufacturer's protocol (Genomic Vision, France). In brief, asynchronous exponentially growing cells were labeled sequentially with the thymidine analogues IdU and CldU for 30 min each. At the end of the CldU pulse, cells were harvested by trypsinization and embedded in low-melting agarose plugs. High molecular weight DNA was isolated using FiberPrep^®^ DNA Extraction Kit (Genomic Vision) from cells embedded in agarose by brief heating to 75°C to melt the agarose followed by agarose digestion. The DNA molecules stretched uniformly and irreversibly attached to a treated glass coverslip (CombiCoverslips, Genomic Vision) using the FiberComb^®^ molecular combing system (Genomic Vision) for immunostaining and fluorescence microscopy. The DNA samples on coverslip were blotted with primary antibodies mouse anti-BrdU (IdU) (BD Biosciences, Cat#347580) and rat anti-BrdU (CldU) (Abcam, Cat#AbC117-7513), followed by goat anti-rat Cy5 (Abcam ab6565) and goat anti-mouse Cy3.5 (Abcam ab6946) secondary antibodies. Origin firing efficiency was determined by counting the fraction of new initiation events—labeled as green only (initiated during the IdU period) or green-red-green (initiated during the IdU period and ongoing in the CIdU period)—among all tracks. Replication elongation efficiency was determined by measuring the mean length of second-label replication tracks in double-labeled tracks to analyze active/ongoing fork rates. Three replicate from each samples were analyzed. A total of 250–450 replication tracks were measured in each sample.

### Statistical analysis

Data are presented as mean ± S.D. Sample numbers (*N* values represent independent biological samples) and experimental repeats are indicated in the figure legends. Statistical significance was determined using Student's *t*-tests and one- or two-way ANOVAs (GraphPad Prism). *P* < 0.05 was considered significant.

## RESULTS

### Nuclear ITCH expression is upregulated in breast cancer

**Figure 1.**
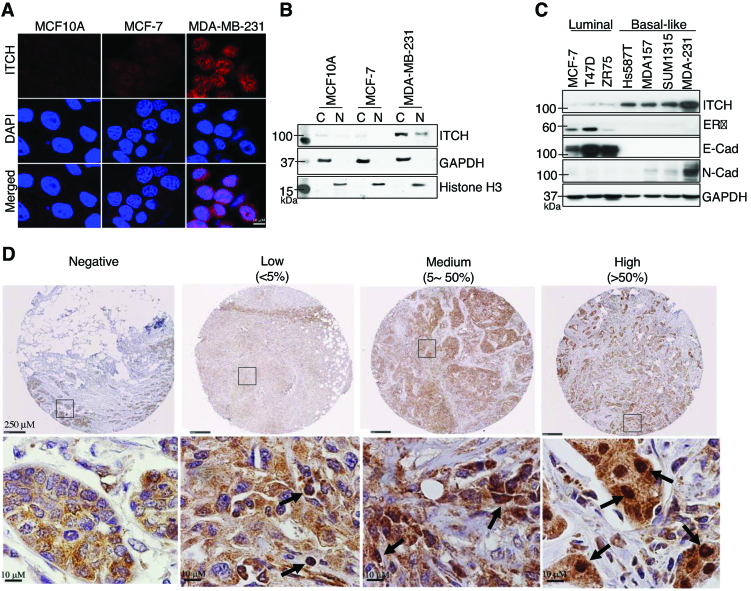
Nuclear ITCH is upregulated in basal-like TNBC. (**A**) Representative IF images of indicated breast cells stained with anti-ITCH antibody (red) and counterstained with DAPI (blue) for cell nuclei. (**B**) Anti-ITCH IB of cytoplasmic (C) and nuclear (N) fractions of indicated cells; GAPDH and H3 are C and N controls, respectively. (**C**) IB of additional luminal and basal-like BC cell lines stained with the indicated antibodies; GAPDH included as a loading control. (**D**) Representative IHC images of a subset of BC tumors, showing negative, low, medium, and high ITCH expression levels stained with anti-ITCH antibody (brown) and counterstained with hematoxylin (blue) for cell nuclei. Arrows indicate ITCH nuclear staining.

We used immunofluorescence (IF), immunohistochemistry (IHC) and immunoblotting (IB) to examine the subcellular localization of ITCH in the following cultured human normal and cancerous breast cell lines with differential tumorigenic and invasive potential: non-tumorigenic mammary epithelial cells (MCF10A); tumorigenic, but non-invasive BC cells (MCF-7); and invasive TNBC cells (MDA-MB-231). MCF10A and MCF-7 cells exhibited low levels of cytoplasmic and nuclear ITCH expression; in contrast, MDA-MB-231 cells expressed both cytoplasmic and nuclear ITCH (Figure 1A and B). We observed similar results in additional cell lines: human mammary epithelial (HMLE) and luminal BC (T47D) cells showed weak cytoplasmic ITCH staining, whereas MDA-MB-157 and HCC1937 TNBC cells showed both cytoplasmic and nuclear expression (Supplementary Figure S1A). To determine whether ITCH expression varied with BC subtype, we performed IB on three luminal (MCF-7, T47D, ZR75) and four basal-like TNBC (Hs578T, MDA-MB-157, SUM1315, MDA-MB-231) cell lines. We found that ITCH protein expression, which correlated with mRNA levels (Supplementary Figure S1B), was higher in basal-like TNBC cells than in luminal cells (Figure 1C). Furthermore, expression of ITCH was positively associated with expression of *N*-cadherin, a marker of metastatic potential, but inversely with E-cadherin (Figure 1C). Compared to ITCH, expression of other HECT family proteins (WWP1, Smurf1, and Nedd4) was elevated but not exclusive to TNBC cells (Supplementary Figure S1C).

To validate our findings in clinical BC specimens, we used IHC to score nuclear ITCH expression in 282 BC cases of varying subtypes, including 136 TNBC cases (Figure 1D). Nuclear ITCH expression was significantly higher in TNBC than in luminal subtypes (*P* < 0.05; Table 1). Nuclear ITCH expression in the 136 TNBC cases also significantly correlated with a histological grade (*P* < 0.01; Table 1). In addition, IHC revealed greater nuclear ITCH expression in lymph nodes containing metastatic BC than in matched primary BC or normal breast tissue (*P* < 0.05; Supplementary Figures S1D and S1E). Altogether, our data present unprecedented evidence for ITCH nuclear localization in TNBC and metastatic BC.

**Table 1.** Quantification of nuclear ITCH levels in indicated BC subtypes and pathohistological grades in TNBC (*italicized*)

Breast cancer subtype	Negative	Low	Medium	High	Total	*P* value*^a^*
Luminal A/B	73	17	28	3	121	<0.05
Her2+	19	6	0	0	25	
Triple-negative (TNBC)	16	24	32	64	136	
*Stage I*	*6*	*4*	*10*	*13*	*33*	<0.01
*Stage II*	*7*	*6*	*5*	*23*	*41*	
*Stage III*	*0*	*1*	*5*	*7*	*13*	
*Stage IV*	*0*	*0*	*0*	*2*	*2*	
*Unknown*	*3*	*13*	*12*	*19*	*47*	
Total	108	47	60	67	282	

^a^ *P* values indicate the relationship between the levels of nuclear ITCH expression and different BC subtypes (top) or clinicopathological grades in TNBC (bottom).

### AKT1-mediated phosphorylation of ITCH at Ser257 drives its nuclear translocation

**Figure 2.**
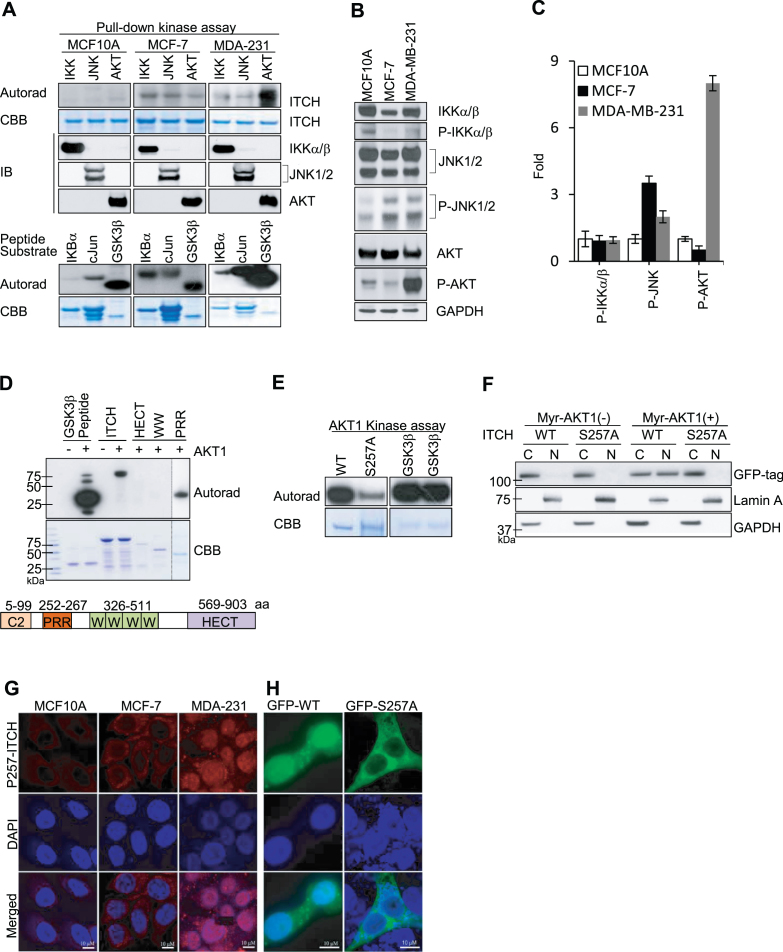
Phosphorylation of ITCH at Ser257 by AKT1 is essential for nuclear translocation in TNBC. (**A**) *In vitro* kinase assay using indicated recombinant proteins as substrates for phosphorylation, visualized by autoradiography (Autorad) and Coomassie Brilliant Blue (CBB) staining for loading controls. (**B**) IB of indicated cell lysates stained with the indicated antibodies and (**C**) Quantification of fold-change relative to expression in MCF10A cells. (D and E) *In vitro* AKT1 kinase assays using (**D**) full-length and truncated ITCH proteins (as shown in schematic; not to scale) and (**E**) WT and S257A ITCH recombinant proteins as substrates. (**F**) IB of cytoplasmic (C) and nuclear (N) compartments in HEK293T cells after transient co-transfection with GFP-WT or GFP-S257 ITCH and Myr-AKT1; GAPDH and Lamin A are C and N controls, respectively. (G–H) Representative IF images of (**G**) indicated cell lines stained with anti-P-S257 ITCH antibody (red) and DAPI, and (**H**) MDA-MB-231 cells transiently transfected with GFP-WT or GFP-S257A ITCH stained with anti-GFP antibody (green) and DAPI (blue). *N* = 3 for IF images.

To determine how ITCH sustains nuclear localization in TNBC, we investigated post-translational protein modifications, which have been identified as important for the regulation of nuclear transport (22). In the presence of cytokines, ITCH is phosphorylated by JNK (c-Jun N-terminal kinase) (14). In addition, we used PHOSPHONET (http://www.phosphonet.ca), a Web-based kinase prediction tool, to identify IκB kinase (IKK) as well as AKT as potential ITCH kinases. To test whether these kinases phosphorylate ITCH in the absence of external stimuli, we used immunoprecipitation (IP) using specific antibodies to pull down endogenous JNK, IKK, and AKT from MCF10A, MCF-7 and MDA-MB-231 cell lysates and tested their kinase activity using recombinant ITCH protein as a substrate. We used IB to confirm pull-down of the relevant kinases (Figure 2A, middle three rows). JNK, IKK and AKT immunocomplexes from MCF-7 and MDA-MB-231 cells phosphorylated ITCH at varying degrees (Figure 2A, upper two rows). None of the kinase immunocomplexes isolated from non-tumorigenic MCF10A cells phosphorylated ITCH. Notably, the AKT immunocomplex from MDA-MB-231 cells, but not MCF-7 or MCF10A cells, strongly phosphorylated ITCH. The activity of IKK, JNK, and AKT immunocomplexes was validated using known peptide substrates (IKBα, cJun and GSK3β, respectively; Figure 2A, bottom two rows). The phosphorylation status of each kinase is also indicative of activation; to measure this, we performed IB of MCF10A, MCF-7, and MDA-MB-231 lysates using relevant phospho-specific antibodies (Figure 2B). Kinase phosphorylation was limited in MCF10A or MCF-7 cells; in contrast, AKT phosphorylation was elevated in MDA-MB-231 cells (Figure 2B and C). Thus, considering its elevated endogenous activity in MDA-MB-231 cells, AKT is an outstanding candidate kinase for ITCH. To verify that ITCH is a direct substrate of AKT, we performed a kinase assay using a purified recombinant form of active AKT1. ITCH was efficiently phosphorylated by recombinant AKT1 (Figure 2D). ITCH contains an N-terminal protein kinase C-related C2 domain, a unique proline-rich region (PRR), four tandem WW domains, and a C-terminal catalytic HECT Ub protein ligase domain(13). We used truncated forms of ITCH as targets and observed that only ITCH containing the PRR domain (amino acids 252–267) was phosphorylated; ITCH containing only the HECT or WW domain was not (Figure 2D). This suggests that the AKT-mediated phosphorylation site is located in the PRR domain of ITCH.

Using MALDI-QIT-TOF-based mass spectrometry (MS) analysis, we identified the Ser (S) 257 residue in the PRR domain as the only residue phosphorylated by AKT1 (Supplementary Figure S2A). An Ala (A) substitution at S257 (S257A) substantially reduced ITCH phosphorylation by AKT1 (Figure 2E). Using a nuclear localization signal (NLS) predictor (23), we demonstrated that S257 was located in the only potential NLS sequence (249-KPSRPPRPSRPPPPTPRR-266) within the ITCH protein. This suggests that S257 phosphorylation confers the ability of ITCH to translocate to the nucleus. To test this, we co-transfected wild-type (WT) or S257A ITCH into cells with a constitutively active form of AKT1 (myristoylated AKT1; myr-AKT1) and compared nuclear localization capacity. In the absence of myr-AKT1, neither WT nor S257A ITCH protein translocated to the nucleus. In the presence of myr-AKT1, WT ITCH translocated to the nucleus, whereas S257A ITCH did not (Figure 2F). We confirmed the role of AKT1 in ITCH nuclear translocation at the single cell level using IF (Supplementary Figure S2B). To further validate the effects of S257 phosphorylation on nuclear localization, we generated a phospho (P)-specific antibody to detect P-S257 ITCH. We used IB to confirm that the antibody detected AKT-mediated phosphorylation at S257 of ectopically expressed ITCH (Supplementary Figure S2C) and endogenous ITCH (Supplementary Figure S2D). IF using anti-P-S257 ITCH antibodies in BC cell lines detected the nuclear localization of P-S257 ITCH protein in MDA-MB-231 cells, but not in MCF10A or MCF-7 cells (Figure 2G). Consistent with this, IF imaging of MDA-MB-231 cells expressing GFP-tagged WT or S257A ITCH revealed that WT but not S257A ITCH had intense nuclear expression (Figure 2H). Altogether, these data demonstrate that AKT-mediated phosphorylation of ITCH at S257 is important for its nuclear localization.

### ITCH nuclear translocation is regulated by a PI3K/AKT-dependent pathway

**Figure 3.**
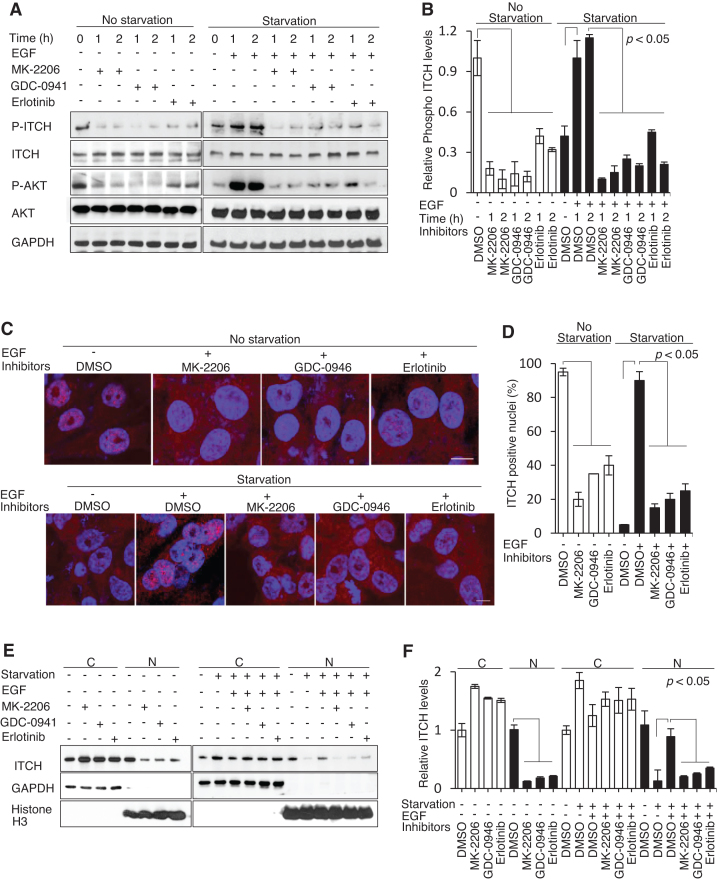
ITCH nuclear translocation is mediated through an EGF/PI3K/AKT-dependent pathway. (**A** and **B**) IB using the indicated antibodies and (**C** and **D**) IF imaging using anti-ITCH antibody (red) and DAPI counterstain (blue) of MDA-MB-231 cells before and after 12–18 h 1% serum starvation with or without prior treatment with MK-2206 (0.1 μM), GDC-0941 (0.05 μM), or Erlotinib (1 μM) following the addition of EGF (10 ng/ml). (**E** and **F**) IB of cytoplasmic (C) and nuclear (N) cellular compartments. *N* = 3. The *P* values indicate significant differences between the experimental groups treated with different inhibitors (MK-2206, GDC-0946, or Erlotinib) and the control groups treated with DMSO under normal or serum-starvation conditions before and after the addition of EGF.

The fact that abundant epidermal growth factor receptor (EGFR or ErbB1) expression and the highly active PI3K-AKT pathway are common and therapeutically targeted in TNBC (24) prompted us to explore whether the EGF/PI3K/AKT signaling pathway can modulate ITCH phosphorylation and nuclear accumulation. EGF/PI3K/AKT signaling can be blocked by treatment with Erlotinib (an EGFR tyrosine kinase inhibitor), GDC-0941 (a pan-PI3K inhibitor), or MK-2206 (a pan-AKT inhibitor) and evaluated by examining downstream phosphorylation of AKT. Using IB analysis, we observed that high levels of endogenous phospho-ITCH, which correlated with high phospho-AKT, were reduced by 2-h treatment with low concentrations of MK-2206 and GDC-0941 and a high concentration of Erlotinib (Figure 3A, left, and Figure 3B). Serum starvation led to reduced levels of phospho-ITCH, again correlating with phospho-AKT, which were induced by the addition of EGF except when prohibited by pre-treatment with MK-2206, GDC-0941, or Erlotinib (Figure 3A, right, and Figure 3B). We confirmed these results at the single cell level using IF imaging, which showed that nuclear ITCH staining was reduced in MDA-MB-231 cells following treatment with MK-2206, GDC-0941 or Erlotinib. Furthermore, following serum starvation, pre-treatment with these inhibitors blocked the nuclear translocation of ITCH upon addition of EGF (Figure 3C and D). Consistent with these findings, we observed the inhibition of nuclear ITCH expression following treatment with MK-2206, GDC-0941 or Erlotinib in subcellular fractionation experiments (Figure 3E and F). These results strongly suggest that the stimulation or inhibition of EGFR to trigger AKT activation or inhibition may modulate ITCH phosphorylation.

### ITCH interacts with and specifically ubiquitinates the linker histone H1.2 at K46

**Figure 4.**
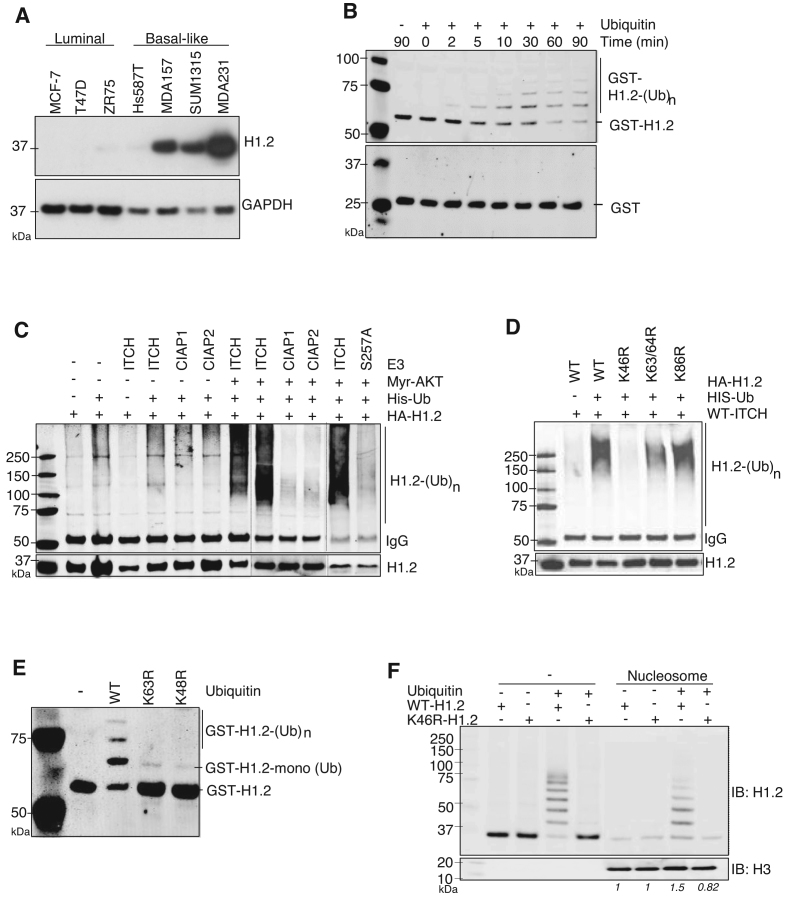
ITCH interacts with and ubiquitinates linker histone H1.2 at K46. (**A**) IB of indicated BC cells stained with anti-H1.2 antibodies; GAPDH included as a loading control. (**B**) *In vitro* ITCH-mediated Ubn of bacterially-expressed recombinant GST alone (bottom) or GST-H1.2 (top) in the absence or presence of ubiquitin. The reaction was terminated at the indicated times and analyzed by IB using anti-GST antibody. (**C** and **D**) *In vivo* Ubn of H1.2 in HEK293T cells co-transfected with the indicated plasmids. Ubiquitinated HA-tagged H1.2 was IP with anti-HA antibodies followed by IB with anti-His antibodies for His-tagged Ubn; WT-, K46R-, K63/64R- or K86R-H1.2 mutants. **(E)** IB using anti-H1.2 antibody to measure *in vitro* ITCH-mediated Ubn of GST-H1.2 with WT or Ub mutants in which lysine is mutated to arginine at K63 or K48. *N* = 3. (**F**) Pull-down of recombinant HA-WT or HA-K46R H1.2 proteins –/+ Ub by biotinylated nucleosomes; IB with indicated antibodies. Relative levels of WT and K46R H1.2 binding to nucleosomes before and after ubiquitination by ITCH, normalized to the pulled down H3 protein levels, are indicated in italics.

To uncover the role of nuclear ITCH, we isolated and identified nuclear proteins that specifically bound to ITCH. First, we performed IP using an anti-ITCH antibody in MDA-MB-231 cell extracts. We then used liquid chromatography coupled with tandem MS (LC−MS/MS) to isolate the ITCH-associated proteins. Among the highly abundant proteins that were pulled down by anti-ITCH antibodies, linker histone protein H1.2 was identified as the most abundant nuclear protein associated with ITCH (Supplementary Figure S3A). Similar to ITCH, H1.2 protein expression was elevated in basal-like TNBC cell lines (Figure 4A) and correlated with mRNA levels (Supplementary Figure S3B). We next used an *in vitro* Ubn assay containing human Ub E1 Enzyme and Ubch7 E2 Enzyme (19) to determine whether H1.2 protein is ubiquitinated by ITCH (Figure 4B). IB of GST-tagged H1.2 revealed a specific high-molecular weight ladder characteristic of poly-Ub chains within two minutes (Figure 4B, top); no laddering was observed using GST alone as a control substrate (Figure 4B, bottom). Omission of Ub protein from the reaction prevented laddering (Figure 4B, top), which confirmed the high-molecular weight species as H1.2-Ub. *In vitro* Ubn assay clearly demonstrated H1.2 to be ITCH’s direct Ubn substrate. We then used transient transfection to show that co-expression of ITCH with histone H1.2 resulted in H1.2-Ub only in cells co-transfected with myr-AKT (Figure 4C). In the presence of truncated ITCH mutants containing only the PRR, WW, or HECT motifs, H1.2 was not ubiquitinated (Supplementary Figure S3C). The RING finger family of E3 ligases, cIAP1 and cIAP2, did not catalyze H1.2 Ubn (Figure 4C). These data identify linker histone protein H1.2 as a specific substrate of ITCH.

Of the seven lysine residues and potential Ubn sites in H1.2 (25), K46, K63, K64 and K86 residues are conserved in both human and mouse. We found that K46 was critical for H1.2 poly-Ubn by ITCH, as a K-to-R single substitution at K46 (K46R-H1.2) completely abolished H1.2-Ub, as did the absence of Ub for WT-H1.2 (Figure 4D). H1.2 Ubn by ITCH at K46 was further verified using Ni-NTA agarose beads to pull down His-Ub followed by Western blot analysis against H1.2 under completely denaturing conditions *in vivo* (Supplementary Figure S3D). In addition, H1.2 poly-Ubn by ITCH was found be dependent on Ub-K48 and Ub-K63 (Figure 4E). To investigate the direct effects of H1.2 K46-Ubn on chromosome binding, we used pull-down assays to assess binding of HA-tagged WT or K46R H1.2 proteins, before and after Ubn by ITCH, to nucleosomes. Purified HA-tagged WT H1.2, but not K46R H1.2, was efficiently polyubiquitinated by ITCH. Prior to Ubn by ITCH, both proteins showed equivalent basal binding to nucleosomes; after Ubn, WT H1.2, but not K46R H1.2, exhibited increased binding affinity for nucleosomes (Figure 4F). The results indicated that ITCH-mediated K46-Ubn is essential for the binding of histone H1.2 to chromatin.

### ITCH-mediated H1.2 K46 Ubn suppresses RNF168/RNF8-dependent 53BP1 foci

**Figure 5.**
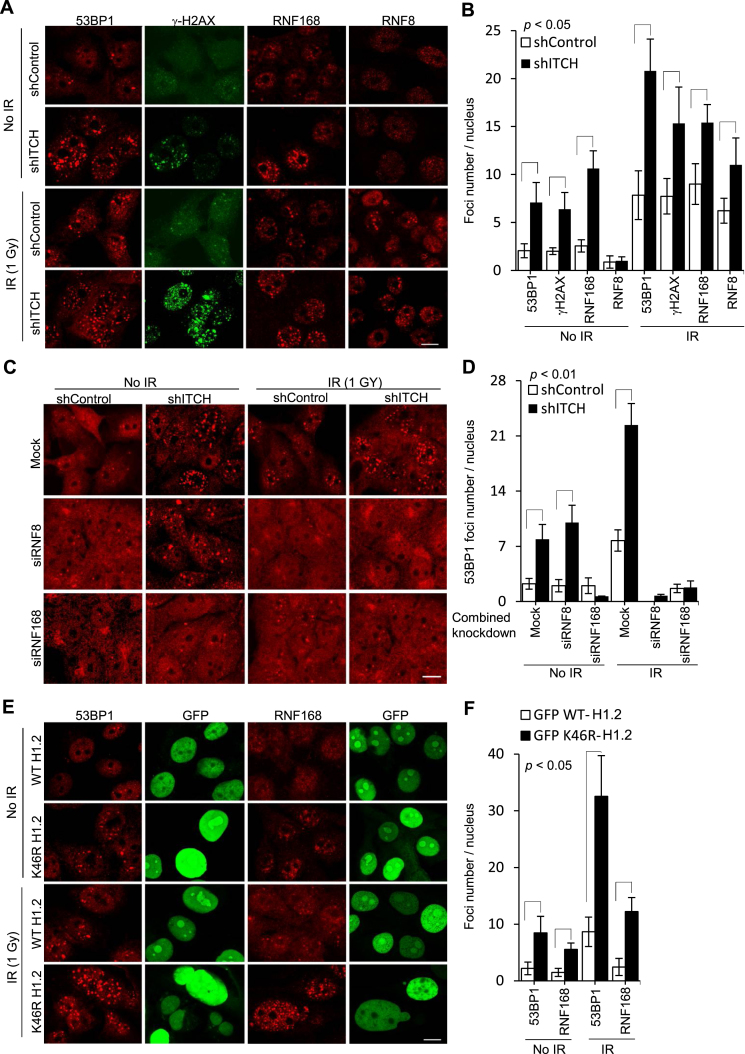
ITCH suppresses RNF168/RNF8-dependent 53BP1 foci formation by H1.2 K46 ubiquination. (**A**, **C** and **E**) Representative IF images of MDA-MB-231 cells, with or without IR exposure (1 Gy, 4 h), using the indicated antibodies against proteins involved in DNA damage and DAPI counterstaining for nuclei; (**B**, **D** and **F**) Quantification of foci/nucleus in 30 nuclei from each group, data are represented as mean ± S.D., *P* values compared between groups indicated with a bracket. (A and B) cells transfected with shITCH for ITCH knockdown or scrambled shRNA control; (C and D) cell transfected with single or combined shRNA knockdown plasmids against ITCH (shITCH), RNF8 (siRNF8), or RNF168 (siRNF168); (E and F) cells overexpressing GFP-WT or GFP-K46R-H1.2, *N* = 3.

Poly-ubiquitinated histone H1 serves as an important signaling intermediate for stimulating the hierarchical sequence of RNF8/RNF168-mediated DDR events at DNA damage sites for 53BP1 foci formation following ionizing radiation (IR) (11). Therefore, we next evaluated whether 53BP1 foci was affected by ITCH and ITCH-mediated H1.2 K46-Ubn (Figure 5). Surprisingly, 53BP1 foci formation was found significantly more evident after ITCH knockdown in shITCH cells than in shControl cells prior to IR exposure. Compared to shControl cells, shITCH cells exhibited severely impaired cell cycle progression (data not shown). Both γH2AX and RNF168 foci were also significantly elevated in cells expressing shITCH, with no significant differences in RNF8 foci formation between groups (*P* < 0.05; Figure 5A and B). After IR exposure, the formation of RNF8, RNF168, 53BP1 and γH2AX foci was significantly elevated in shITCH cells compared to shControl cells (*P* < 0.01; Figure 5A and B). Unlike ITCH knockdown, before IR, single RNF168 or RNF8 knockdown exerted no significant effects in 53BP1 foci formation (*P* < 0.01; Figure 5C and D). We also assessed whether the heightened formation of 53BP1 foci shITCH cells was RNF168- or RNF8-dependent by examining 53BP1 foci formation in MDA-MB-231 cells after knockdown of ITCH in combination with RNF8 or RNF168 knockdown. The formation of accumulated intrinsic 53BP1 foci in shITCH cells before IR was significantly abolished by simultaneous RNF168 knockdown, but was unaffected by simultaneous RNF8 knockdown (*P* < 0.001; Figure 5C and D, two left panels). After IR, however, RNF8 and RNF168 knockdown almost completely suppressed IR-induced 53BP1 foci in both shControl and shITCH cells (*P* < 0.001; Figure 5C and D, two right panels). Taken together, these data suggest that ITCH deficiency-induced 53BP1 foci accumulation is dependent on RNF168 and RNF8.

In addition, BRCA-1 and RAP80 foci accumulated to a greater extent in shITCH cells than in shControl cells (Supplementary Figures S4A and S4B), and depletion of ITCH resulted in the accumulation of 53BP1 foci in a BRCA-1 deficient TNBC cell line, HCC1937 (Supplementary Figures S4C and S4D). We also observed an increase in γH2AX accompanied by ITCH nuclear translocation in MDA-MB-231 cells after exposure to IR or the chemotherapeutic drug hydroxyurea (HU) (Supplementary Figures S4E-S4H). Constitutively high γH2AX foci formation in shITCH cells was further elevated by IR. The γH2AX foci induced by IR or HU decreased in shControl cells within 24 or 4 h after exposure, respectively, but were sustained in shITCH cells (Supplementary Figures S4E-S4H). In addition to greater accumulation of γH2AX foci, we observed greater accumulation of Phospho-ATR (Ser428) and Phospho-ATM (Ser1981) induced by HU and IR, respectively, in shITCH cells compared to shControl cells (data not shown). These data strongly indicate that ITCH helps to suppress the DDR to external damage.

MDA-MB-231 cells after treatment of AKT inhibitor MK-2206 was able to recapitulate DDR phenotypes of ITCH knockdown (Supplementary Figure S5). In addition to reduced nuclear ITCH, significantly elevated 53BP1 and γH2AX foci were found in MDA-MB-231 cells after MK-2206 treatment. Low but detectable levels of nuclear ITCH were also found in MCF-7 cells. Only slight changes in the levels of nuclear ITCH and 53BP1/γH2AX foci were observed in MCF-7 after MK-2206 treatment. The results suggest a potential role of nuclear ITCH in the DNA damage response.

We next investigated whether ITCH-mediated K46 H1.2 Ubn can impact 53BP1 foci formation. MDA-MB-231 cells transgenically overexpressing GFP-tagged WT H1.2 or Ubn-defective K46R H1.2 were generated (Supplementary Figure S4I). The average number of intrinsic 53BP1 foci in individual K46R-H1.2-overexpressing cells was around 4-fold higher than in WT H1.2-overexpressing cells (*P* < 0.01; Figure 5E and F). After IR exposure, 53BP1 foci formation increased in both cell types, but remained 4-fold higher in K46R-H1.2 cells than WT-H1.2 cells. RNF168 foci formation was also elevated in K46R-H1.2 cells to a greater extent than in WT-H1.2-overexpressing cells prior to and after IR. These data strongly indicated that ITCH-mediated H1.2 K46 Ubn is important for preventing 53BP1 accumulation.

### ITCH biochemically antagonized RNF168 and RNF8 in polyubiquitination of histone H1.2

**Figure 6.**
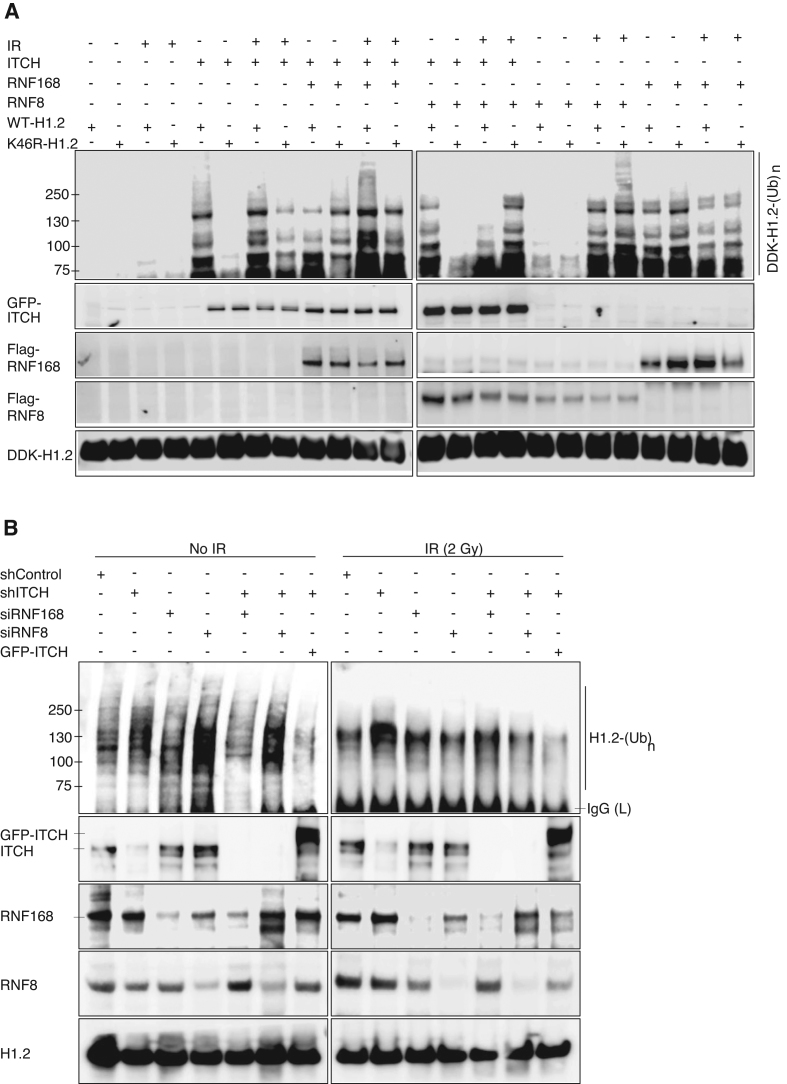
ITCH biochemically competes with RNF168 and RNF8 to polyubiquitinate histone H1.2. (**A**) *In vivo* Ubn of WT- or K46R-H1.2 in HEK293T cells co-transfected with single or combined GFP-tagged ITCH, Flag-tagged RNF168, or Flag-tagged RNF8 plasmids as indicated, with or without IR exposure (2 Gy, 4 h). Ubiquitinated DDK-tagged H1.2 was IP with anti-DDK antibodies and detected via IB with anti-His antibodies. (**B**) IB of Ubn levels of endogenous H1.2 in MDA-MB-231 after transfection with single or combined scrambled shRNA (shControl), ITCH (shITCH), RNF168 (siRNF168), RNF8 (siRNF8) knockdown or GFP-ITCH plasmids, with or without IR exposure (2 Gy, 4 h) using Ub antibody after IP with H1.2 antibody. Endogenous protein levels were examined using the indicated antibodies. *N* = 3.

The observation of opposing actions of ITCH and RNF168/RNF8 on 53BP1 foci formation at the single cell level (Figure 5) prompted us to examine whether ITCH can biochemically suppress RNF8/RNF168-mediated H1.2 poly-Ubn. We first assessed the level of H1.2 Poly-Ubn after ITCH co-transfected with RNF168 or RNF8, along with WT- or K46R-H1.2 expression plasmids in 293T cells (Figure 6A). In contrast with cells transfected with ITCH, RNF168, and RNF8 plasmids, control cells transfected only with H1.2 showed little to no detectable H1.2 ubiquitination before or after exposure to IR. Prior to IR, RNF8 did not ubiquinate H1.2. Interestingly, RNF168 ubiquinated WT-H1.2 and to an even greater extent K46R-H1.2 (Figure 6A, left panel). After IR exposure, RNF8 ubiquitinated WT-H1.2 and to a greater extent K46R-H1.2. In contrast, basal levels of RNF168-mediated H1.2 Ubn were not significantly elevated by IR (Figure 6A, right panel). The results suggest that RNF168 is primarily responsible for endogenous H1.2 Ubn whereas RNF8 is primarily responsible for IR-induced H1.2 Ubn. Both RNF168 and RNF8 elicited higher Ubn levels of K46R-H1.2 compared to WT-H1.2, suggesting that Ubn of H1.2 by both E3 ligases occurs at a site apart from K46. Most importantly, co-transfection with WT ITCH expression plasmids reduced RNF168- and RNF8-mediated H1.2 Ubn (Figure 6A). The data indicates that ITCH antagonizes RNF168- and RNF8- mediated H1.2 Ubn.

To confirm the negative relationship between ITCH and RNF168/RNF8 for H1.2 Ubn, endogenous H1.2 Ubn was evaluated from MDA-MB-231 cells after knockdown of ITCH and/or RNF168 or RNF8. shRNA transfection to knockdown ITCH (shITCH) resulted in elevation of endogenous H1.2 Ubn compared to transfection with a scrambled shRNA control (shControl) prior to and after IR (Figure 6B, left and right panels, respectively). Elevated H1.2 Ubn induced by ITCH knockdown was reduced significantly when combined with RNF168 knockdown prior to IR (Figure 6B, left panel) or RNF8 knockdown after IR (Figure 6B, right panel). Re-expressed ITCH reduced the levels of H1.2 Ubn prior to and after IR in ITCH knockdown cells. The results strongly suggest the ITCH can negatively regulate RNF168 prior to IR and RNF8 after IR for H1.2 Ubn.

### ITCH makes cells resistant to replication stress and DNA damage

**Figure 7.**
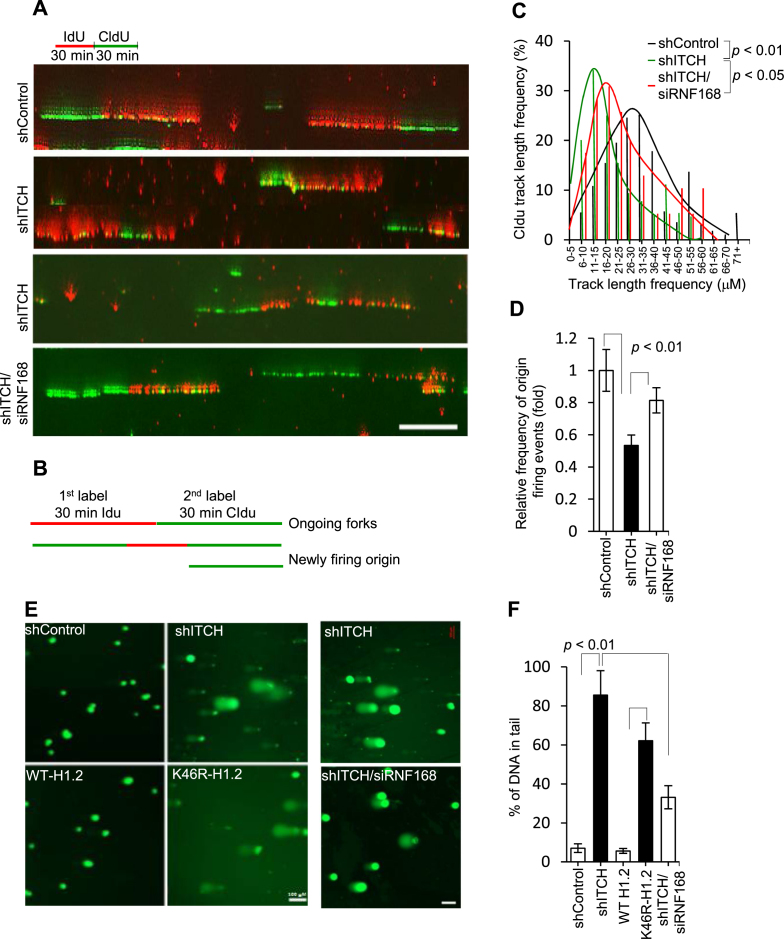
ITCH makes cells resistant to replication stress and DNA damage. (**A**) MDA-MB-231 cells transfected with single or combined scrambled shRNA (shControl), ITCH (shITCH), or RNF168 (siRNF168) were labeled consecutively with IdU (red) and CldU (green), for 30 min each, before isolating and stretching DNA for IF, as illustrated in the schematic. Representative images of dual-labeled fibers are shown. (**B**) Schematics showing ongoing tracks sequentially labeled with IdU (red) and CIdu (green). Origin firing events are labeled in green only (initiated during the IdU period) or green-red-green (initiated during the IdU period and ongoing in the CIdU period). Tracks with stalled/collapsed forks during the IdU period are labeled in red only. The mean CldU tract length distributions of ongoing tracts (**C**) and the fold change of new origin firing events defined by these two track types among all labeled tracks (**D**) are shown for three independent experiments in which 200–450 tracks/experiments were analyzed for shControl, shITCH, or combined shITCH and siRNF168 cells. shControl was given an arbitrary value of 1.0. (**E)** Representative IF images (N = 3) of cell nuclei assessed for single-stranded and double-stranded breaks using the comet assay at alkaline pH from the agarose-embedded nuclei of MDA-MB-231 cells after transnfection with shControl, shITCH, or shH1.2 re-expressing WT- or K46R-H1.2 as well as combined shITCH and siRNF168. Cells were stained with SYBR-green after separation of DNA fragments by electrophoresis. Scale bar, 100 μm. (**F**) Quantification of tail moment detected using comet assays from a total of 90–100 cells in triplicate were analyzed per group. Data are represented as mean ± S.D.

53BP1 functions as a tumor suppressor that inhibits the invasion and growth of BC cells (26). Persistent 53BP1 foci are indicative of cells subjected to replication stress and DNA damage (27). To directly evaluate replication at the single molecule level, we conducted DNA fiber assays to analyze incorporation of labeled nucleotides into newly synthesized DNA fibers after consecutive 30 min pulses of 5-iodo-2′-deoxyuridine (IdU) and 5-chloro-2′-deoxyuridine (CldU) (Figure 7A and B). Replication fork progressivity can be determined by measuring the mean length of CIdU second pulse-label replication tracks in double-labeled tracks. Relative CIdU segments were decreased significantly in shITCH cells compared to shControl cells (Figure 7C). Additionally, numbers of newly firing origins were reduced in shITCH cells (Figure 7D). These data strongly suggested that obstructed replication fork progression might be resulted from persistent 53BP1 foci by ITCH knockdown. Alkaline comet assays, in which the percentage of tail DNA relative to total DNA is indicative of the levels of DNA damage present in an individual cell, confirmed the data that DNA damage was significantly augmented in ITCH knockdown cells or H1.2 knockdown cells expressing K46R-H1.2 compared to shControl or H1.2 knockdown cells expressing WT-H1.2, respectively (Figure 7E and F). RNF168 knockdown in shITCH cells significantly improved replication fork progression (Figure 7A–D) and reduced DNA damage (Figure 7E and F), as well as γH2AX foci accumulation (Supplementary Figures S6A and S6B). Furthermore, ITCH depleted and reconstituted K46R-H1.2 cells (Supplementary Figure S4J) were sensitized to IR (Supplementary Figure S7A) and HU (Supplementary Figure S7B) treatment. Taken together, these data supported the concept that the ITCH-H1.2 axis confers resistance to replication stress and DNA damage.

**Figure 8.**
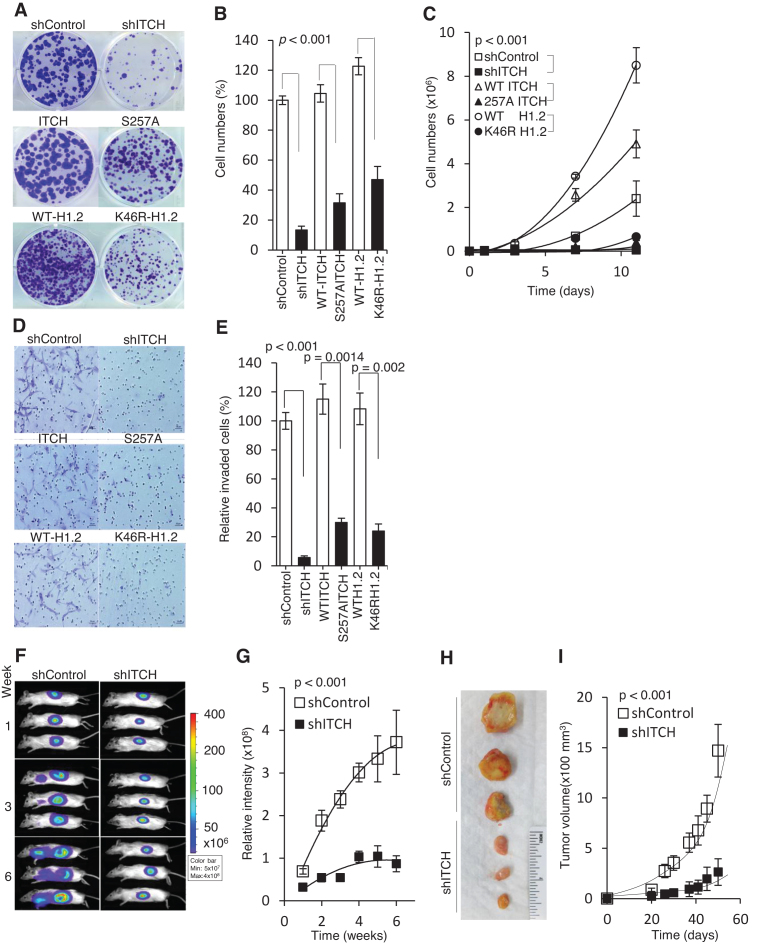
Disruption of the ITCH-H1.2 pathway limits cell growth and migration in MDA-MB-231 cells. (**A**) Representative image (N = 3) of clonogenic assay of MDA-MB-231 cells stably expressing shITCH or shControl; GFP-tagged WT ITCH, S257A ITCH; H1.2 shRNA knockdown reconstituted with GFP-tagged WT H1.2 or K46R H1.2. Cells were seeded at 500 cells. Colonies were fixed and stained with crystal violet two weeks later. (**B**) Quantitative measurement of clonogenic assays in (A) from triplicate experiments. (**C**) 500 cells from each group were plated in triplicate on day 0 and counted every 48 h. (**D**) Representative images of matrigel invasion assays performed in the above cell groups, stained with 0.5% methylene blue. (**E**) Quantitative measurement of invasive cells in (D). (**F**) Representative bioluminescence images of mice (*N* = 12) taken at the indicated times. Images show tumor growth following injection of shITCH or shControl MDA-MB-231 cells into the fourth mammary fat pad of NSG mice; (**G**) Quantitative analysis of tumor bioluminescence signals over time (n = 12). (**H**) Representative images of tumor growth in NSG mice (*N* = 12) at week 6 following xenograft; (**I**) Time course of tumor growth in NSG mice. *P* values compared between groups indicated with a bracket. Data represented as mean ± S.D.

Consistent with this notion, ITCH-mediated K46 H1.2 Ubn may promote cancer growth and metastasis. Thus, we tested whether disruption of the ITCH-H1.2 pathway limits cell viability and migration in MDA-MB-231 cells. Transgene S257A ITCH overexpressed in MDA-MB-231cells was also generated (Supplementary Figure S4K). Compared to shControl, WT ITCH, and WT-H1.2 cells, respectively, shITCH, S257A ITCH, and K46R-H1.2 cells showed significantly reduced colony formation (*P* < 0.001; Figure 8A and B), proliferation (*P* < 0.001; Figure 8C), and invasion (*P* < 0.05; Figure 8D and E). To demonstrate the effects of ITCH knockdown *in vivo*, we used tumor xenograft mouse models of MDA-MB-231 cells. shITCH MDA-MB-231 tumors showed significantly reduced growth and invasiveness relative to shControl tumors (*P* < 0.001; Figure 8F–I). Thus, we provide evidence that the ITCH-mediated K46 H1.2 cascade is required for tumor growth and metastatic progression.

## DISCUSSION

Elevated PI3K/AKT oncogenic signaling is considered a hallmark of carcinomas (17). AKT can be activated by PI3K or PI3K-like kinase (PIKK) family members: ataxia-telangiectasia mutated (ATM), ataxia-telangiectasia and Rad3-related (ATR), and DNA-dependent protein kinase catalytic subunit (DNA-PKcs) (28). In turn, activated AKT has been shown to be directly involved in inhibition of homologous-recombination (29) and non-homologous-end-joining (30) repair pathways to facilitate tumorigenesis. Our data demonstrate that ITCH serves as a signal transmitter in the EGFR/PI3K/AKT pathway to negatively regulate RNF168/RNF8-dependent DDR in rapidly replicating TNBC cells. We showed that nuclear translocation and S257 phosphorylation of ITCH are induced by IR and HU, and that IR-induced ITCH and P-S257 ITCH can be reduced more efficiently when AKT is inhibited. This suggests that AKT may mediate ITCH nuclear translocation through PIKK family members after exposure to IR. ITCH was previously shown to be phosphorylated by ATM kinase in response to DNA damage (31). The possibility that the same DNA damage that activates ATM also directly modulates ITCH nuclear localization cannot be ruled out at this point.

Following the EGFR/PI3K/AKT pathway, we identify that H1.2 proteins can be efficiently and specifically ubiquitinated by ITCH at K46. However, the function of ITCH-mediated H1.2 Ubn at K46 suppressed rather than enhanced 53BP1 foci formation. Unlike the RNF8/RNF168-dependent DDR Ubn of H1.2, which is Ub-K63-specific (11), ITCH-mediated poly-Ubn of H1.2 was observed to be Ub-K63- and Ub-K48- dependent. We therefore propose a hypothesis that ITCH-mediated H1.2 K46 ubiquitination causes the formation of a steric hindrance to prevent full access of RNF8/RNF168. Unlike ITCH using one E2 UbcH7, H1.2 Ubn by RNF8 was previously shown to require two E2 enzymes, UBCH5c and UBC13 E2 enzymes (11). Additionally, the specific RNF8/RNF168- dependent H1.2 Ubn site was not clearly defined. Clearly comparing binding affinity to H1.2 between ITCH, RNF8, and RNF168 and mapping of RNF8/RNF168- dependent ubiquitination sites will help to understand how ITCH-mediated K46 Ubn would prevent RNF8/RF68 ubiquitylation of H1.2.

Linker histone H1.2 might not be the only ITCH’s Ubn substrate to regulate DDR. The ITCH-mediated K46 residue in H1.2 is well conserved and identified in related family members H1.3 and H1.4 (32). Therefore, in addition to H1.2, Histone H1.3 and H1.4 can be ITCH’s potential substrates to media DDR. However, the model establishing linker H1 as the target of RNF8 Ubn to mediate IR-induced 53BP1 foci accumulation has been recently challenged. L3MBTL2, a putative polycomb group (PcG) protein, was identified as a key target of RNF8 following DNA damage (33). The specific sites of DNA damage-induced RNF8-mediated Ubn have not yet been identified for either protein. Identification of the Ubn sites will provide valid evidence as specific substrate of RNF8.

We observed that ITCH knockdown caused elevation of endogenous 53BP1 and γH2AX foci formation, which was dependent on RNF168 but not RNF8. Furthermore, RNF168 but not RNF8 can efficiently ubiquitinate H1.2 in the absence of IR. The localization of endogenous 53BP1 and γH2AX foci may mark the sites of eroded telomeres (34), as well as clustered unrepaired DSB in tumors (35). The elevation of 53BP1 foci occurred throughout the genome, including telomeric DNA, and thus may impair the proliferative capacity of tumor cells. RNF168 has been shown to directly recruit 53BP1 to DNA damage sites independently of the γH2AX-MDC1-RNF8 signaling axis (36). For example, RNF168 can interact with 53BP1 in an IR-independent manner and facilitate the formation of a protein complex containing RNF168, 53BP1 and MDC1 (36). Interaction of RNF168 with 53BP1 and its E3 ligase activity was shown to be required for the initial recruitment and retention of 53BP1 to DNA damage sites. It is possible that the H1.2 Ubn by RNF168 that we observed in this study may help to recruit the 53BP1/MDC1 protein complex to chromatin. Thus, enhancement of RNF168 binding and Ubn to H1.2 after ITCH deficiency might increase recruitment and retention of 53BP1 to DNA damage sites and lead to induction of spontaneous DDR signaling induced by replication stress.

53BP1 functions as a tumor suppressor and plays a critical role in cell cycle checkpoint and DNA repair activities. Accumulation of 53BP1 is associated with human TNBC and BRCA1-related BCs (37) and contributes to their resistance to poly(ADP-ribose) polymerase inhibitors (38). These data indicate that loss of 53BP1 accumulation might be a common mechanism for advanced tumors to evade oncogenic and chemotherapeutic agent-induced DNA damage stress. We observed that treatment with the AKT inhibitor MK-2206 reduced nuclear ITCH accumulation and enhanced 53BP1/γH2AX foci formation in MDA-MB-231 cells, but not in MCF-7 cells in which ITCH expression was very low. Taken together, these data suggest that ITCH can function as downstream effector of AKT for controlling the replication stress response and cell growth and migration via the 53BP1 DNA damage response pathway. Several ITCH-related HECT family members (e.g., Smurf1, Smurf2, WWP1 Nedd4) and their protein substrates (e.g., Notch1, SMAD2, PTEN, p53) are involved in tumorigenesis (13) and may serve as promising targets for cancer therapies. ITCH knockdown sensitized MDA-MB-231 cells to replication stress and slowed cell growth and migration. The abundance of total and nuclear ITCH proteins appears to be important for MDA-MB-231 cells to maintain genome stability and counteract replication stress. Because low levels of nuclear ITCH protein were found in MCF-7 and MCF10A cells (Figure 1A and B), ITCH may not be as important for counteracting replication stress in these cell types as it is for MDA-MB-231 cells. Indeed, after siRNA knockdown of ITCH, cytotoxicity was reduced in MCF-7 cells and nearly absent in MCF10A cells (data not shown). These data suggest that the effect of ITCH knockdown may be selective for TNBC cells. Furthermore, depending on the cellular milieu, ITCH may act as a proapototic or prosurvival factor, which may pose a substantial challenge to drug development. ITCH inhibitors will need to be specific, selected not only based on its enzymatic catalytic site. We expect that the work presented here will facilitate the development of specific ITCH inhibitors that can block AKT-mediated ITCH S257 phosphorylation and subsequent H1.2 K46 Ubn for a potential cancer treatment regimen.

For further details regarding Methods used in this work, see SUPPLEMENTAL DATA.

## Supplementary Material

Supplementary Data
