# Supplementary material for: ITCH nuclear translocation and H1.2 polyubiquitination negatively regulate the DNA damage response
Source: Nucleic Acids Res. 2018 Dec 4;47(2):824–42. doi: 10.1093/nar/gky1199 (PMC6344871; doi:10.1093/nar/gky1199)
Supplement: Supplementary Data [file gky1199_supplemental_files.pdf]

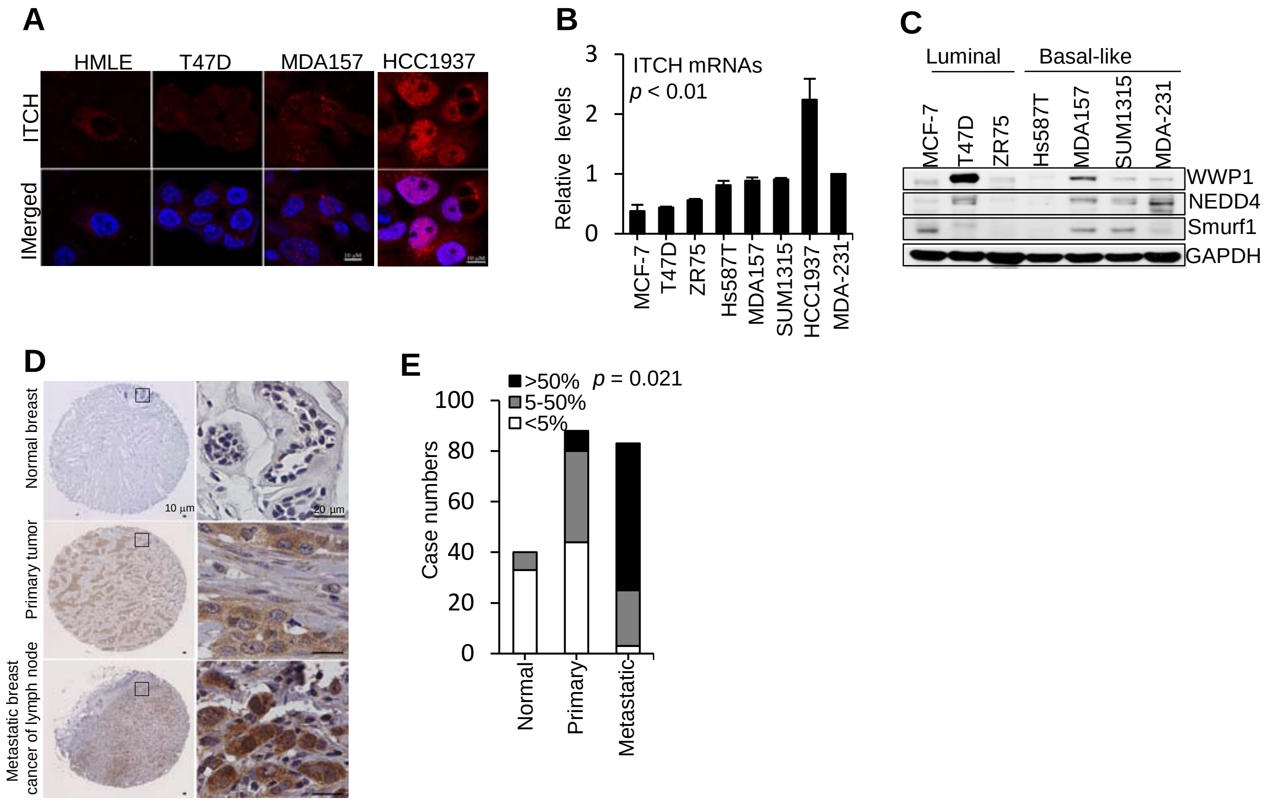

Figure S1. Nuclear ITCH is upregulated in basal-like TNBC

(A) Representative confocal IF images of the intracellular localization of ITCH in human mammary epithelial cells (HMLE), luminal BC cells (T47D), and TNBC cells (MDA-MB-157, shown as MDA157); ITCH antibody stain shown in red and nuclei counterstained blue with DAPI.

(B) qPCR analysis of normalized ITCH mRNA in the indicated cell lines.

(C) IB analysis of WWP1, NEDD4, and Smurf1 in the indicated cell lines.

(D) Representative IHC images of ITCH staining in normal breast, primary BC tumor, and matched lymph node tissue containing metastatic BC; ITCH antibody stain shown in brown and nuclei counterstained blue with hematoxylin.

(E) Nuclear ITCH staining quantified according to sample type. ITCH protein expression was scored as negative, low (<5%), medium (5-50%), and high (>50%) (see Supplemental Experimental Procedures) in normal breast (N=40) , primary BC tumor (N=88), and matched lymph node tissue (N=82) containing metastatic BC. *p* value indicates that the case numbers of nuclear ITCH staining (>50%) in lymph node tissue containing metastatic BC significantly different from that of other groups either at the low (<5%) or medium (5-50%) levels.

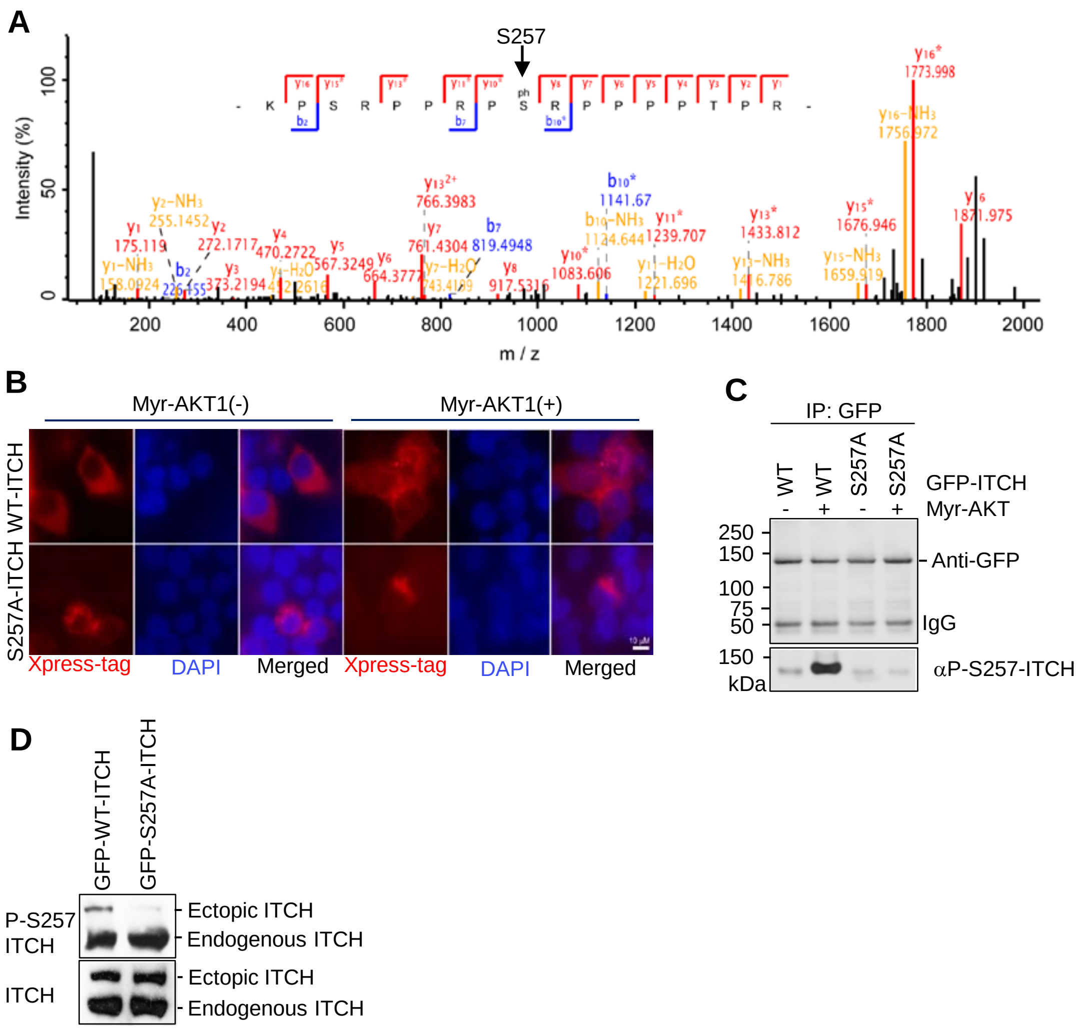

Figure S2. S257 phosphorylation by AKT1 is essential for its nuclear translocation

(A) Identification of AKT1 phosphorylation sites on ITCH. The AKT1 phosphorylation site in ITCH was investigated by examining AKT1-phosphorylated recombinant ITCH using MALDI-QIT-TOF-based MS analysis. MS identified the tryptic peptide 249-KPSRPPRPpSRPPPPTPR-265. Peptide sequencing is indicated by the B-ion (blue) and Y-ion (red) fragment series, and the identified peptide was matched to ITCH by electronic database search.

(B) Representative IF images of intracellular localization of Xpress-tagged (red) WT or S257A ITCH in 293T cells 48 h after transient transfection with Myr-AKT(+) or empty vector (-). DAPI counterstain (blue) for cell nuclei identification.

(C) IB analysis using anti-GFP or anti-phospho-S257 (P-S257) ITCH antibody on GFP-tagged WT or S257A ITCH immunoprecipitated from the protein lysates of 293T cells co-transfected with Myr-AKT or empty vector.

(D) IB analysis using anti-ITCH or anti- P-S257 ITCH antibody on GFP-tagged WT or S257A ITCH after transient transfection into MDA-MB-231 cells.

A

Proteins IP by anti-ITCH antibodies

Accession #	Peptide	Protein name	M.W.
IPI00455315	23	ANXA2 Isoform 1 of Annexin A2	38.60
IPI00007188	9	SLC25A5 ADP/ATP translocase 2	32.85
IPI00027626	9	CCT6A T-complex protein 1 subunit zeta	58.02
IPI00186290	16	EEF2 Elongation factor 2	95.34
IPI00941747	8	CANX Calnexin	67.57
IPI00942979	19	TKT Transketolase	67.88
IPI00217465	3	**HIST1H1.2 Histone H1.2**	21.36
IPI00553185	12	CCT3 T-complex protein 1 subunit gamma	60.53
IPI00221222	2	RNA polymerase II transcriptional coactivator p15	14.40
IPI00018206	9	GOT2 Aspartate aminotransferase, mitochondrial H2AFV	47.52
IPI00398805	1	histone H2A.V isoform 4	6.89
IPI00985068	2	CDK1 Uncharacterized protein	21.72
IPI00306383	1	Secretory carrier-associated membrane protein 3 HNRNPD	35.20
IPI00965952	5	Uncharacterized protein	23.08
IPI00002966	5	HSPA4 Heat shock 70	94.33
IPI00006666	2	SLC16A3 Monocarboxylate transporter 4	49.47
IPI00008215	2	ME1 NADP-dependent malic enzyme	64.15
IPI00012442	4	G3BP1 Ras GTPase-activat	52.16
IPI00024403	2	CPNE3 Copine-3	60.13
IPI00025273	7	GART Trifunctional purine biosynthetic protein adenosine-3	107.77
IPI00027834	2	HNRNPL Heterogeneous nuclear ribonucleoprotein L	64.13
IPI00166205	5	KRT78 Isoform 2 of Keratin, type II cytoskeletal 78 KRT13	45.02
IPI00171196	10	Isoform 3 of Keratin, type I cytoskeletal 13	45.87
IPI00329633	5	TARS Teonyl-tRNA synthetase, cytoplasmic	83.43
IPI00386975	2	DSC1 Isoform 1B of Desmocollin-1	93.83
IPI00843876	5	TNPO1 Isoform 2 of Transportin-1	101.31
IPI00877999	3	SNORD43;RPL3 35 kDa protein	34.97
IPI00917777	12	U5 small nuclear ribonucleoprotein component isoform b	105.38
IPI00924906	2	ASNS Asn synthetase [glutamine-hydrolyzing] isoform c	54.82
IPI00947363	4	ATP-dependent RNA helicase DDX5	67.76
IPI00983581	4	SYNCRIP SYNCRIP protein (Fragment)	50.65
IPI01010870	11	ITCH Uncharacterized protein	86.50
IPI00019912	7	HSD17B4 Peroxisomal multifunctional enzyme type 2	79.69
IPI00643908	2	Hepatoma-derived growth factor	23.72
IPI00217413	1	DHX29 ATP-dependent RNA helicase DHX29	155.23

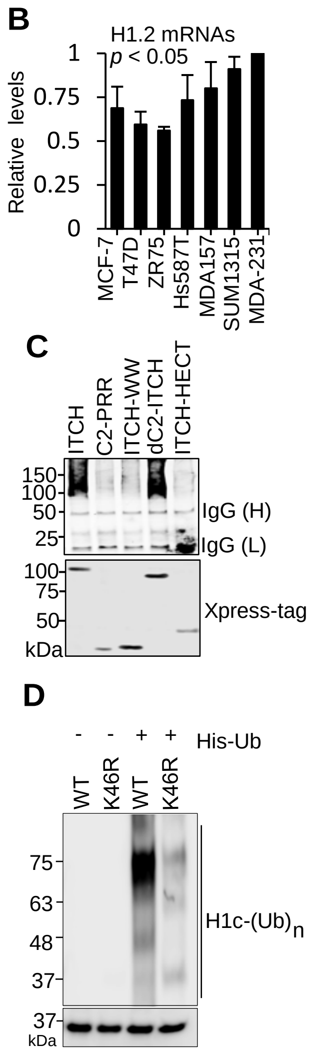

Figure S3. ITCH interacts with and ubiquitinates the linker histone H1.2 at K46

(A) Partial list of ITCH-associated polypeptides purified by co-IP with anti-ITCH antibodies and analyzed by LC-MS/MS. Calculation includes background subtraction using co-IP with non-specific mouse monoclonal IgG antibodies from MDA-MB-231 cells. Linker histone protein H1.2 (red) was identified as the top nuclear protein associated with ITCH.

(B) qPCR analysis of histone H1.2 mRNAs in the indicated cell lines.

(C) In vivo ubiquitination (Ubn) of H1.2 in 293T cells after transient transfection with Xpress-tagged full length ITCH (ITCH), C2 and PRR domains (C2-PRR), WW domain (ITCH-WW), deleted in C2 domain (dC2-ITCH), or HECT domain (ITCH-HECT), along with Myr-AKT1, HA-H1.2, and His-ubiquitin (Ub) plasmids. Histone Ubn was visualized by IP with anti-HA antibodies and IB with anti-His antibodies. Expression of full length or deleted ITCH constructs in cells was examined by IB using anti-Xpress antibodies.

(D) Ubn of H1.2 at K46 by ITCH in vivo by pull-downed His-tagged ubiquitinated H1.2 from 293T cells after transfection with indicated plasmids along with Myr-AKT1 and His-Ub plasmids using Ni-NTA agarose. Pull-downed his-Ubiquitin and his-Ubiquitin-conjugated proteins beads were then analyzed by IB using anti-HA antibodies. IB of the total lysates using anti-HA antibodies for HA-tagged H1.2 for the loading control (bottom).

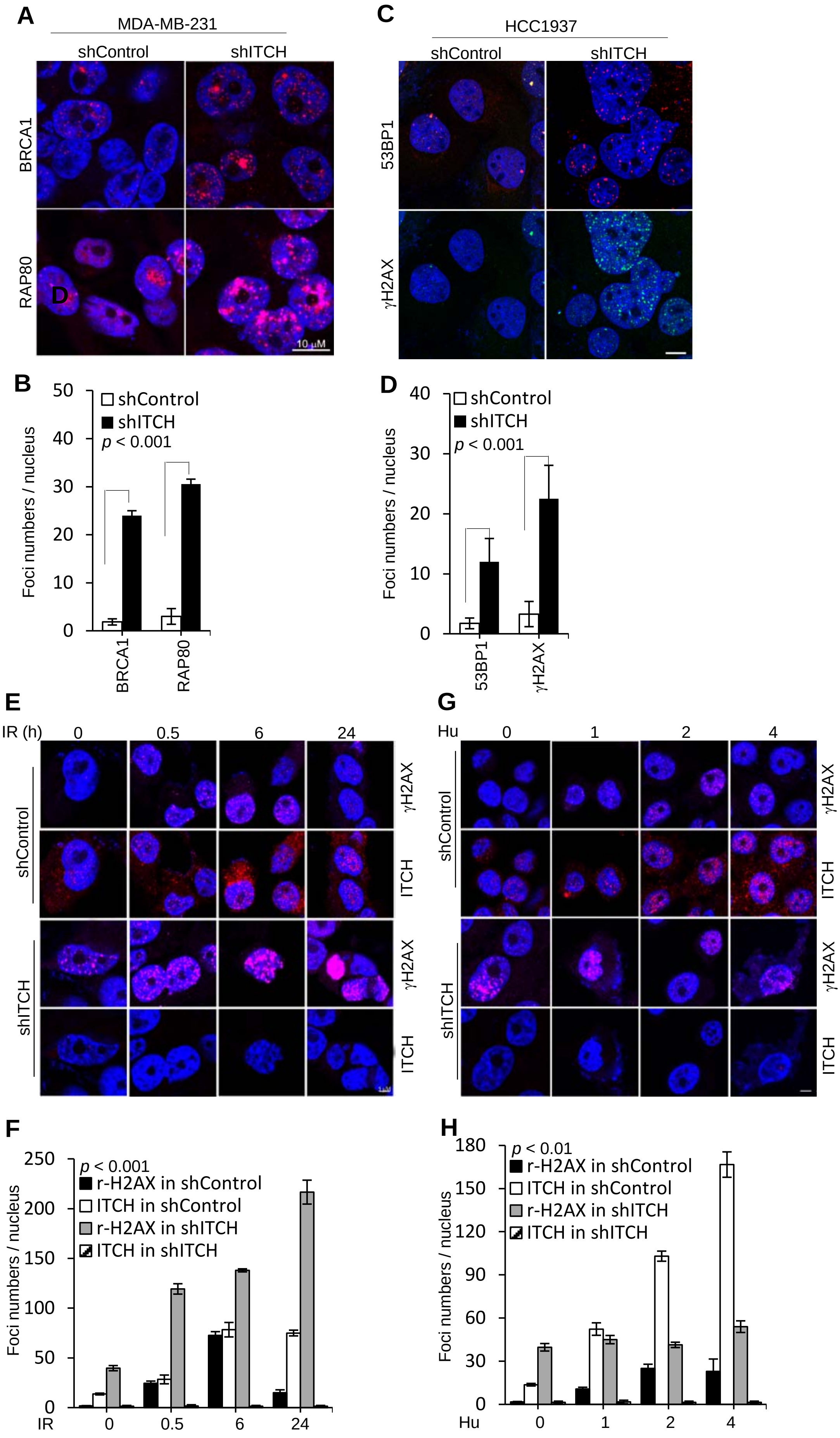

Supplementary Figure S4

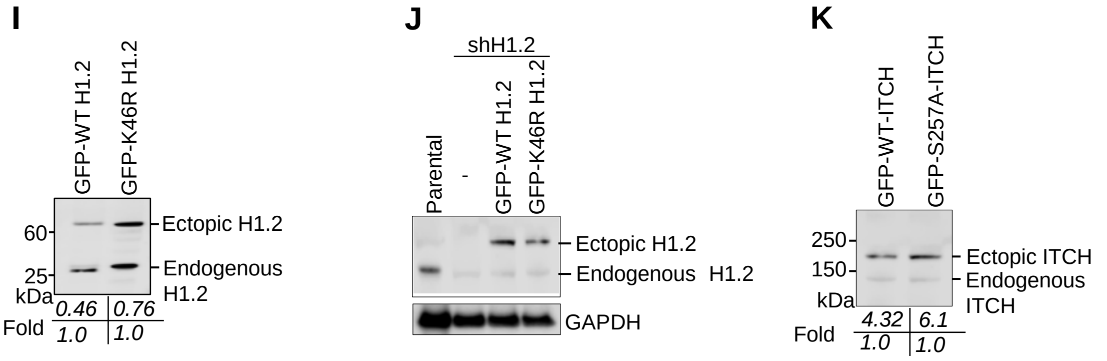

Figure S4. ITCH knockdown results in elevated 53BP1 and γ-H2AX in MDA-MB-231 and HCC1937 cells

(A, C) Representative IF images (N=3) using indicated antibodies against proteins involved in DNA damage in MDA-MB-231 (A) or HCC1937 cells (C) after ITCH (shITCH) or scrambled shRNA (shControl) knockdown.

(B, D) Quantification of indicated protein foci averaged from 30 nuclei in each group.

(E-H) Representative IF confocal images (N=3) of MDA-MB-231 ITCH knockdown (shITCH) or scrambled shRNA (shControl) cells before and after IR (2 Gy) (E) and Hu (Hydroxyurea, 0.5 mM) (G) at the indicated times with the indicated antibodies and the quantification (F-H) from a total of 30 nuclei from each group.

(I-K) IB analysis of the indicated transgene levels in MDA-MB-231 cells after stably expressing GFP-tagged WT or K46R H1.2 using anti-H1.2 antibodies (I); in H1.2-knockdown MDA-MB-231 cells after stably expressing GFP-WT or GFP-K46R H1.2 using anti-H1.2 antibodies (J); in MDA-MB-231 cells after stably expressing GFP-tagged WT and S257A ITCH using anti-ITCH antibodies (K). The relative protein levels between ectopic and endogenous proteins in each cell line are indicated in italic numbers.

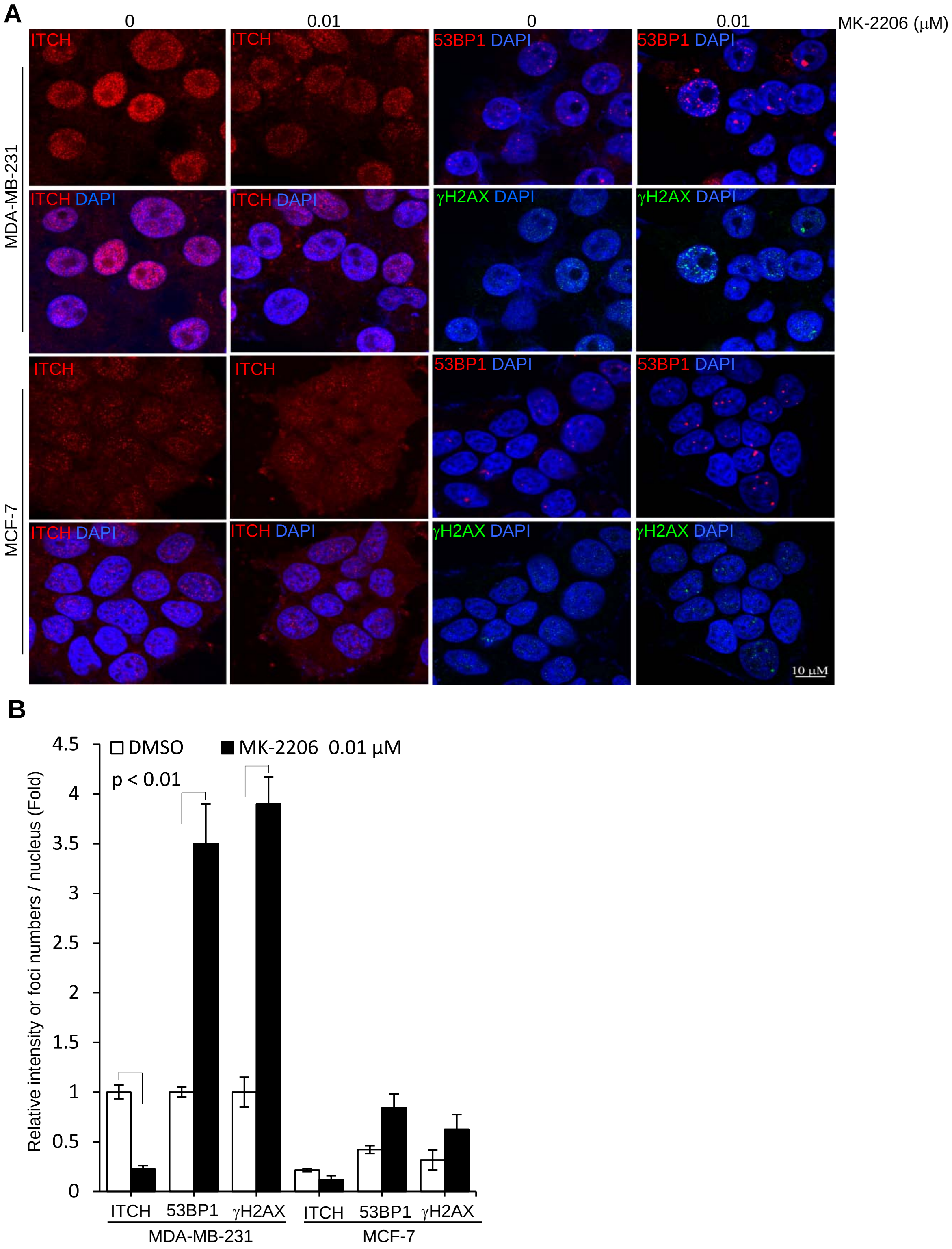

Figure S5. ITCH, 53BP1 and γH2AX foci accumulation in MDA-MB-231and MCF-7 cells after treatment of AKT inhibitor MK-2206

(A) Representative IF images (N=3) using antibodies against ITCH, 53BP1, and γH2AX involved in DNA damage in MDA-MB-231 and MCF-7 after treatment of MK-2206 and counterstained with DAPI (blue) for cell nuclei.

(B) Quantification of relative intensity or foci numbers / nucleus in 30 nuclei from each group, data are represented as mean ± S.D., p values compared between groups indicated with a bracket. The fold change (Fold) in ITCH intensity and foci numbers/nucleus of 53BP1 and γH2AX was relative to MDA-MB-231 treated with DMSO, which was given an arbitrary value of 1.0.

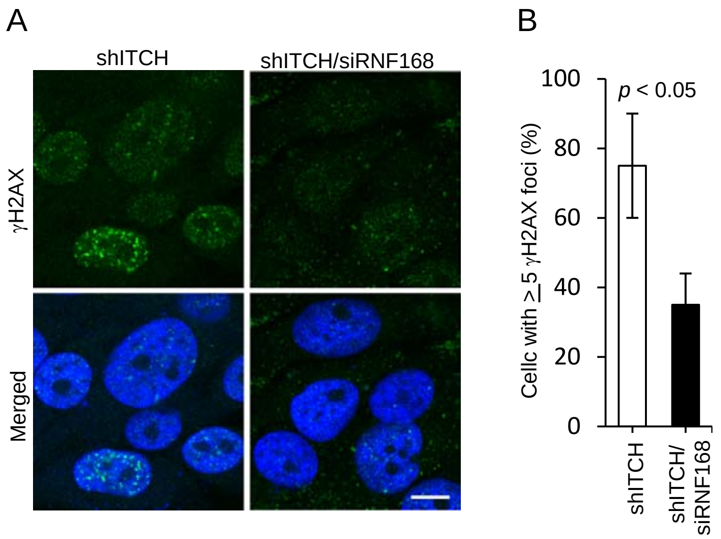

Figure S6. RNF168 knock-down reduced ITCH deficiency-induced γH2AX in MDA-MB-231 cells

(A) Representative IF images (N=3) using antibodies against γH2AX in MDA-MB-231 cells transfected with single or combined shRNA knockdown plasmids against ITCH (shITCH) or RNF168 (siRNF168).

(B) Quantification of foci/nucleus in 30 nuclei from each group, data are represented as mean ± S.D.

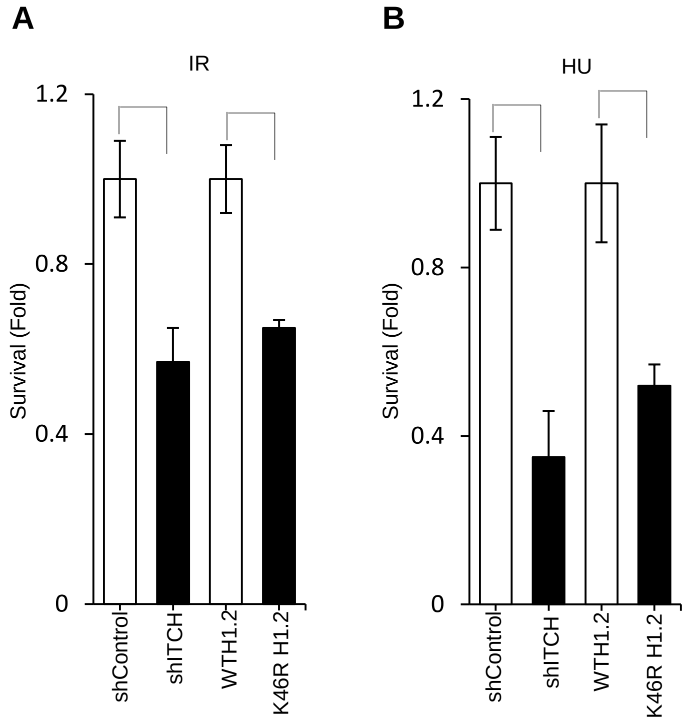

Figure S7. Disruption of the ITCH-H1.2 axis sensitizes MDA-MB-231 cells to IR and HU

Cell viability demonstrated as fold change of 2500 MDA-MB-231 cells stably expressing shControl, shITCH , or H1.2 shRNA reconstituted with GFP-tagged WT H1.2 or K46R H1.2 plated in triplicate and counted 48 h after IR (2 GY)(A) or HU (0.5 mM)(B) treatment.

Supplementary Table I: Reagent resource

REAGENT or RESOURCE	SOURCE	IDENTIFIER
Antibodies		
GAPDH	Cell Signaling Technology, Inc	Cat# 5174
Histone H3	Cell Signaling Technology, Inc	Cat# 4499
E-Cadherin	Cell Signaling Technology, Inc	Cat# 3195
N-Cadherin	Cell Signaling Technology, Inc	Cat# 4061
Vimentin	Cell Signaling Technology, Inc	Cat# 5741
IKKα/β	Cell Signaling Technology, Inc	Cat# 2682
JNK1/2	Cell Signaling Technology, Inc	Cat# 2684
pan-AKT	Cell Signaling Technology, Inc	Cat# 4672
Phospho-IKKα/β (Ser176/180)	Cell Signaling Technology, Inc	Cat# 4691
Phospho-SAPK/JNK (Thr183/Tyr185)	Cell Signaling Technology, Inc	Cat# 2697
Phospho-Akt (Ser473)	Cell Signaling Technology, Inc	Cat# 4668
ATM	Cell Signaling Technology, Inc	Cat# 4060
ATR	Cell Signaling Technology, Inc	Cat# 2873
Phospho-ATM (Ser1981)	Cell Signaling Technology, Inc	Cat# 2790
Phospho-ATR (Ser428)	Cell Signaling Technology, Inc	Cat# 4526
H2AX	Cell Signaling Technology, Inc	Cat# 2853
His-tag	Cell Signaling Technology, Inc	Cat# 7631
HA-tag	Cell Signaling Technology, Inc	Cat# 9991
IKKγ	BD Biosciences	Cat# 611306
JNK1/2	BD Biosciences	Cat# 4499
ITCH	BD Biosciences	Cat# 61198
BrdU (B44, IdU)	BD Biosciences	Cat# BD347580
HA-tag (for IP)	Roche	Cat# 11867423001
ERα	Bethyl Laboratories, Inc	Cat# A300-498A-T
MDC-1	Bethyl Laboratories, Inc	Cat# A300-051-A
BRCA1	Bethyl Laboratories, Inc	Cat# A301-378A-T
H1c	Abcam	Cat# ab181973
BU1/75 (ICR1	Abcam	Cat# ab6326
53BP1	Novus Biologicals	Cat# NB100-304SS
GFP-tag	Novus Biologicals	Cat# NB600-308
RNF8	Dr. Xiachun Yu's laboratory	
RAP80	Dr. Xiachun Yu's laboratory	
RNF168	R&D Systems	Cat# AF7217
Ubiquitin (FK2)	Enzo Life Sciences	Cat# BML-PW8810-0100
γ-H2AX	EMD Millipore	Cat# 05-636
DNA G quadruplex (G4), clone 1H6	EMD Millipore	Cat# MABE1126
DDK-tag	OriGene Technologies, Inc.	Cat# TA50011-100
Alexa Fluor 555 conjugated donkey anti-mouse (Ifor IdU)	Life Technology	Cat# A-31570
Alexa Fluor 488 conjugated donkey anti-Rat IgG (H+L) Highly Cross (For CIdU)	Life Technology	Cat# A-21208

Alexa Fluor 488-, 568- or 350- conjugated goat anti-mouse or rabbit IgG (H+L) antibodies	Life Technology	Cat# A31570
Biological Samples		
100 cases of breast cancer tissue array with normal tissue as control (BR1002a)	US Biomax	Cat# BR1002a
50 cases of invasive ductal carcinoma and matched metastatic invasive ductal carcinoma of lymph node from breast (BR1005)	US Biomax	Cat# BR1005
282 invasive breast cancers with clinical data including ER/PR/HER2 status, grades, and stages	Markey Cancer Center Biospecimen Tissue and Procurement Shared Resource Facility (P30CA177558) in University of Kentucky	
Chemicals, Peptides, and Recombinant Proteins		
GSK3β peptide (10-25 aa)	Cell Signaling Technology, Inc.	Cat# 9237
GST-tagged H1c	Novus Biologicals	Cat# H00003006-P01-2μg
53BP1 tudor-like region	Cayman Chemical	Cat# 14073
Mononucleosomes, Human Biotinylated	EpiCypher	Cat# 16-0006
Ubiquitin E1 Enzyme	R&D Systems	Cat# E-304
E2 Conjugating Enzyme<br>Ubiquitin	R&D Systems	Cat# E2-640
Ubiquitin	R&D Systems	Cat# U-530
GST-IKBα ((1-54 a.a.)	This paper	
GST-cJun (1-77 a.a.)	This paper	
MK-2206	Cayman Chemical	Item# 11593
GDC-0941	Cayman Chemical	Item# 11600
Erlotinib	Selleckchem	Cat# S7786
QuikChange II Site-Directed Mutagenesis Kit	Agilent Technologies	Cat# 200524
FiberPrep® DNA Extraction Kit	Genomic Vision)	
Deposited Data		
Experimental Models: Cell Lines		
MCF10A	ATCC	Cat# ATCC® CRL-10317™
MCF-7	ATCC	Cat# ATCC® HTB-22™
MDA-MB-231	ATCC	Cat# ATCC® HTB-26™
Human mammary epithelial (HMLE)	Laboratory of Peter Zhou; Cancer Cell 23, 316–331, March 18, 2013	Cat# ATCC® PCS-600-010™
T47D	Laboratory of Peter Zhou; Cancer Cell 23, 316–331, March 18, 2013	Cat# ATCC® HTB-133™
MDA-MB-157	Laboratory of Peter Zhou; Cancer Cell 23, 316–331, March 18, 2013	Cat# ATCC® HTB-24™
Hs578T	Laboratory of Peter Zhou; Cancer Cell 23, 316–331, March 18, 2013	ATCC® HTB-126
SUM-1315	Laboratory of Peter Zhou; Cancer Cell 23, 316–331, March 18, 2013	
HCC1937	Laboratory of Xiaochun Yu, J Cell Sci. 2013 May 1;126	

Recombinant DNA		
pcDNA3.1	Invitrogen	
pET-32a (+) vector	Novagen	Cat# 69015-3
DDK-tagged H1.2	OriGene Technologies	Cat# RC201249
pRK5-HA-Ubiquitin-K63	Addgene (Cambridge, MA)	Cat# 17606
pRK5-HA-Ubiquitin-K48	Addgene (Cambridge, MA)	Cat# 17605
pRK5-HA-Ubiquitin-K29	Addgene (Cambridge, MA)	Cat# 22903
HA-H1c	Laboratory of Xiaochun Yu, J Cell Sci. 2013 May 1;126	
pEGFP-C1	Clontech; Laboratory of Yanzhong Yang	
Sequence-Based Reagents		
MISSION shITCH RNA lentivirus	Sigma-Aldrich	TRCN0000002090 (shITCH-1); TRCN0000002088 (shITCH-2); TRCN0000002089 (shITCH-3); TRCN0000002087 (shITCH-4); TRCN0000010680 (shITCH-5)
MISSION® Lentiviral Controls	Sigma-Aldrich	Cat# SHC004V
Software and Algorithms		
ZEISS Microscope Software ZEN	ZEISS	
Image Studio Lite Ver5.2	LI-COR	
GraphPad Prism7	GraphPad Software, Inc.	http://www.graphpad.com/
